# Supplementary material for: DamID identifies targets of CEH-60/PBX that are associated with neuron development and muscle structure in Caenorhabditis elegans
Source: PLoS One. 2020 Dec 11;15(12):e0242939. doi: 10.1371/journal.pone.0242939 (PMC7732058; doi:10.1371/journal.pone.0242939)
Supplement: S1 Fig — In both young adults (A) and L2 larvae (B), high correlation values are observed within dam::ceh-60 samples (≥0.67 in YA, ≥0.65 in L2). Correlation between gfp::dam and dam::ceh-60 is also high (≥0.52 in YA, ≥0.51 in L2). (DOCX) [file pone.0242939.s001.docx]

# A

gfp::dam_1

gfp::dam_2

gfp::dam_3

dam::ceh-60_1

dam::ceh-60_2

dam::ceh-60_3

**0.68**

**0.71**

**0.53**

**0.57**

**0.57**

**0.79**

**0.56**

**0.53**

**0.52**

**0.53**

**0.52**

**0.52**

**0.62**

**0.64**

**0.66**

dam::ceh-60_1

dam::ceh-60_2

dam::ceh-60_3

gfp::dam_1

gfp::dam_2

gfp::dam_3

0.0 0.1 0.2 0.3 0.4 0.5 0.6 0.7 0.8 0.9 1.0

# B

dam::ceh-60_1

dam::ceh-60_2

dam::ceh-60_3

gfp::dam_2

gfp::dam_3

gfp::dam_1

**0.66**

**0.73**

**0.51**

**0.5**

**0.6**

**0.75**

**0.53**

**0.51**

**0.52**

**0.53**

**0.53**

**0.58**

**0.59**

**0.56**

**0.59**

dam::ceh-60_1

dam::ceh-60_2

dam::ceh-60_3

gfp::dam_1

gfp::dam_2

gfp::dam_2

0.0 0.1 0.2 0.3 0.4 0.5 0.6 0.7 0.8 0.9 1.0

**S1 Fig: Spearman’s rank-order correlation coefficient for *dam::ceh-60* and *gfp::dam* samples.** In both young adults (A) and L2 larvae (B), high correlation values are observed within *dam::ceh-60* samples (≥0.67 in YA, ≥0.65 in L2). Correlation between *gfp::dam* and *dam::ceh-60* is also high (≥0.52 in YA, ≥0.51 in L2).
