# Supplementary material for: DamID identifies targets of CEH-60/PBX that are associated with neuron development and muscle structure in Caenorhabditis elegans
Source: PLoS One. 2020 Dec 11;15(12):e0242939. doi: 10.1371/journal.pone.0242939 (PMC7732058; doi:10.1371/journal.pone.0242939)
Supplement: S1 Table — Start and stop represent the genomic positions on the specified chromosome (chr) of the specified open reading frame. Candidate gene targets are sorted by genomic position (i.e. chromosome number and start position). Log2FC is calculated as the log2 value of the ratio of average number of dam::ceh-60 reads over average number of gfp::dam reads. (DOCX) [file pone.0242939.s002.docx]

**S1 Table: 872 candidate gene targets of CEH-60 identified in young adult animals through DamID.** Start and stop represent the genomic positions on the specified chromosome (chr) of the specified open reading frame. Candidate gene targets are sorted by genomic position (*i.e.* chromosome number and start position). Log_2_FC is calculated as the log_2_ value of the ratio of average number of *dam::ceh-60* reads over average number of *gfp::dam* reads.

| **Gene** | **chr** | **Start** | **Stop** | **Log_2_FC** |
| --- | --- | --- | --- | --- |
| *Y65B4BL.6* | I | 520109 | 521554 | 0.910 |
| *ZC123.4* | I | 841742 | 846763 | 1.707 |
| *Y95B8A.6* | I | 882918 | 890194 | 1.717 |
| *him-19* | I | 889771 | 895105 | 1.361 |
| *C54G6.3* | I | 996045 | 1005066 | 1.252 |
| *npr-23* | I | 1062293 | 1065269 | 1.202 |
| *Y48G8AL.10* | I | 1136371 | 1154386 | 1.719 |
| *Y92H12BL.5* | I | 1365309 | 1376409 | 1.241 |
| *dyb-1* | I | 1483083 | 1490494 | 0.885 |
| *mab-20* | I | 1644588 | 1651394 | 0.772 |
| *tln-1* | I | 1721592 | 1740670 | 1.390 |
| *C53H9.2* | I | 1832897 | 1834764 | 1.217 |
| *rpl-27* | I | 1834881 | 1835439 | 1.088 |
| *emr-1* | I | 1842445 | 1843227 | 3.885 |
| *Y71G12A.4* | I | 1885205 | 1894905 | 1.257 |
| *Y37E3.22* | I | 2069884 | 2070023 | 0.964 |
| *ptr-12* | I | 2587527 | 2595294 | 0.924 |
| *Y23H5A.10* | I | 2614878 | 2615027 | 0.901 |
| *F32B5.6* | I | 2668160 | 2672434 | 0.771 |
| *F32B5.7* | I | 2672940 | 2682797 | 0.966 |
| *Y71F9B.21* | I | 2703263 | 2703458 | 1.831 |
| *lpd-6* | I | 3144410 | 3147793 | 1.810 |
| *cdap-2* | I | 3319540 | 3330673 | 0.919 |
| *fbxa-203* | I | 3371769 | 3373834 | 2.046 |
| *W05F2.4* | I | 3374776 | 3407189 | 1.366 |
| *C50F2.2* | I | 3881816 | 3888313 | 1.722 |
| *abf-1* | I | 3886111 | 3887080 | 1.465 |
| *C50F2.4* | I | 3888331 | 3891598 | 1.592 |
| *C50F2.5* | I | 3895764 | 3897478 | 1.314 |
| *fkb-5* | I | 3898046 | 3899840 | 2.032 |
| *pbs-4* | I | 3908747 | 3909701 | 1.168 |
| *lrp-2* | I | 3935272 | 3953991 | 0.918 |
| *aars-2* | I | 3983171 | 3986915 | 1.166 |
| *unc-73* | I | 3999348 | 4031739 | 1.099 |
| *unc-89* | I | 4035702 | 4090884 | 1.546 |
| *nab-1* | I | 4229577 | 4236039 | 0.899 |
| *W09C3.2* | I | 4711414 | 4713572 | 1.133 |
| *M04F3.5* | I | 4773818 | 4788358 | 0.833 |
| *glo-2* | I | 4836187 | 4844966 | 1.158 |
| *pdxk-1* | I | 4848425 | 4850717 | 1.089 |
| *F21F3.4* | I | 4896003 | 4897541 | 3.201 |
| *dip-2* | I | 4934118 | 4949332 | 1.278 |
| *pat-10* | I | 5018770 | 5020166 | 2.091 |
| *C46H11.6* | I | 5020748 | 5023210 | 1.735 |
| *kin-32* | I | 5086317 | 5093793 | 0.945 |
| *pfkb-1.1* | I | 5131288 | 5134101 | 1.826 |
| *osr-1* | I | 5200258 | 5203504 | 1.148 |
| *C32E12.4* | I | 5230549 | 5242607 | 1.649 |
| *B0261.8* | I | 5271265 | 5272037 | 2.418 |
| *F55A12.13* | I | 5357541 | 5357785 | 1.735 |
| *F59A3.2* | I | 5512048 | 5514557 | 1.692 |
| *tns-1* | I | 5583407 | 5607529 | 1.067 |
| *hil-5* | I | 5774585 | 5775610 | 2.335 |
| *cpb-3* | I | 5788634 | 5791752 | 2.488 |
| *C34G6.9* | I | 5879139 | 5879491 | 1.161 |
| *C34G6.11* | I | 5879340 | 5879554 | 1.161 |
| *pgp-2* | I | 5886613 | 5895970 | 1.233 |
| *trak-1* | I | 6122590 | 6126851 | 1.012 |
| *tmed-3* | I | 6562699 | 6563660 | 1.205 |
| *ZC581.7* | I | 6660436 | 6662315 | 1.422 |
| *pqn-20* | I | 6791025 | 6797470 | 1.472 |
| *H27M09.7* | I | 6847134 | 6847308 | 0.777 |
| *H27M09.6* | I | 6847139 | 6847303 | 0.777 |
| *C48B6.13* | I | 6893303 | 6893447 | 1.583 |
| *ubql-1* | I | 7021946 | 7029076 | 1.600 |
| *tofu-5* | I | 7069440 | 7070728 | 1.500 |
| *unc-15* | I | 7376604 | 7383197 | 1.710 |
| *unc-13* | I | 7422785 | 7454088 | 1.034 |
| *F26A3.1* | I | 7640338 | 7641767 | 1.134 |
| *ego-1* | I | 7650418 | 7656336 | 0.816 |
| *F30F8.1* | I | 7832692 | 7835453 | 1.744 |
| *pck-2* | I | 7866136 | 7872080 | 0.922 |
| *jph-1* | I | 7946186 | 7951593 | 1.802 |
| *lin-10* | I | 8109746 | 8119314 | 1.169 |
| *D1081.16* | I | 8459041 | 8459254 | 2.676 |
| *D1081.15* | I | 8459050 | 8459331 | 2.676 |
| *rskn-1* | I | 8573855 | 8580157 | 0.911 |
| *K07A12.5* | I | 8693451 | 8695159 | 1.371 |
| *DY3.8* | I | 8758156 | 8759032 | 2.403 |
| *tin-13* | I | 8759204 | 8759788 | 1.493 |
| *lmn-1* | I | 8762986 | 8765633 | 2.658 |
| *ztf-26* | I | 8853686 | 8861274 | 0.993 |
| *F32H2.13* | I | 8987095 | 8987231 | 1.299 |
| *prom-1* | I | 9287437 | 9289770 | 1.062 |
| *skr-2* | I | 9469704 | 9470633 | 1.129 |
| *F30A10.16* | I | 9500624 | 9500823 | 0.979 |
| *C41G7.9* | I | 9515002 | 9516776 | 1.189 |
| *C04F12.5* | I | 9690079 | 9690557 | 1.398 |
| *C16C2.8* | I | 9722096 | 9722380 | 0.872 |
| *tba-1* | I | 9785778 | 9787624 | 1.090 |
| *K10C3.4* | I | 9853658 | 9863492 | 1.520 |
| *T24D1.3* | I | 9958782 | 9960155 | 1.398 |
| *cfi-1* | I | 9994731 | 9999928 | 1.576 |
| *Y106G6D.1* | I | 10107393 | 10109000 | 2.703 |
| *F37D6.4* | I | 10488648 | 10489569 | 2.071 |
| *C35E7.3* | I | 10836985 | 10838877 | 1.248 |
| *tag-296* | I | 10933810 | 10935712 | 1.535 |
| *Y53H1B.2* | I | 11292662 | 11294197 | 1.395 |
| *col-66* | I | 11559446 | 11560565 | 2.552 |
| *clec-101* | I | 11560637 | 11564387 | 1.832 |
| *sri-11* | I | 11695339 | 11697943 | 1.563 |
| *fbxa-122* | I | 12006911 | 12008040 | 0.962 |
| *Y47H10A.5* | I | 12095777 | 12097987 | 1.003 |
| *F14B6.t1* | I | 12229309 | 12229380 | 2.209 |
| *F41D3.6* | I | 12264521 | 12266267 | 1.581 |
| *K11D2.5* | I | 12513516 | 12518694 | 1.028 |
| *K11D2.4* | I | 12518787 | 12527906 | 1.299 |
| *atp-1* | I | 12652277 | 12654646 | 1.652 |
| *efsc-1* | I | 12978839 | 12982452 | 1.122 |
| *W05H12.2* | I | 13411594 | 13412879 | 1.712 |
| *W05H12.4* | I | 13411814 | 13412423 | 1.681 |
| *soem-1* | I | 13491425 | 13493317 | 1.303 |
| *Y87G2A.13* | I | 13598618 | 13603393 | 0.980 |
| *linc-1* | I | 13652524 | 13652727 | 1.159 |
| *rpl-31* | I | 13652939 | 13653704 | 1.147 |
| *Y6B3B.1* | I | 13692929 | 13702954 | 1.460 |
| *fbxa-93* | I | 13829662 | 13831607 | 1.130 |
| *W04A8.2* | I | 13840706 | 13841846 | 0.880 |
| *ZC334.12* | I | 14028997 | 14029212 | 1.585 |
| *C49A1.5* | I | 14237359 | 14241188 | 1.085 |
| *C49A1.10* | I | 14241898 | 14244452 | 3.695 |
| *clec-115* | I | 14246228 | 14247314 | 2.045 |
| *fbxa-103* | I | 14287872 | 14295898 | 1.216 |
| *F49B2.3* | I | 14311009 | 14312871 | 2.403 |
| *Y105E8A.2* | I | 14347514 | 14358326 | 1.986 |
| *unc-95* | I | 14381344 | 14384737 | 2.052 |
| *zoo-1* | I | 14529531 | 14546634 | 1.073 |
| *lev-11* | I | 14621173 | 14631254 | 1.285 |
| *pbs-5* | I | 14719167 | 14720477 | 1.295 |
| *hmp-2* | I | 14738882 | 14742657 | 0.871 |
| *rps-26* | I | 14759918 | 14760654 | 1.544 |
| *smp-1* | I | 14807467 | 14815725 | 0.881 |
| *metl-17* | I | 14839856 | 14843128 | 0.813 |
| *aex-5* | I | 14850054 | 14855827 | 2.154 |
| *unc-54* | I | 14855901 | 14863573 | 2.167 |
| *klu-1* | I | 14975419 | 14980586 | 1.156 |
| *F23F1.2* | II | 34204 | 35262 | 0.918 |
| *C24H12.2* | II | 436867 | 438748 | 1.026 |
| *W08F4.12* | II | 582111 | 584403 | 1.644 |
| *F54D12.11* | II | 1393488 | 1394105 | 1.077 |
| *K05F6.12* | II | 1545925 | 1546991 | 3.869 |
| *fbxb-12* | II | 1753526 | 1754439 | 1.072 |
| *F07E5.9* | II | 2076291 | 2080526 | 5.639 |
| *sup-9* | II | 2684669 | 2686977 | 1.653 |
| *T05A8.5* | II | 2731319 | 2735250 | 1.068 |
| *fbxb-108* | II | 2764964 | 2767230 | 2.520 |
| *lido-9* | II | 3000768 | 3002411 | 0.876 |
| *ZC239.6* | II | 3209478 | 3211181 | 3.587 |
| *F02E11.7* | II | 3267663 | 3271505 | 1.109 |
| *C46E10.1* | II | 3724225 | 3725909 | 2.102 |
| *pqn-48* | II | 4038868 | 4043070 | 1.701 |
| *rpn-11* | II | 4043059 | 4044330 | 0.815 |
| *C16A11.3* | II | 4229551 | 4230686 | 2.211 |
| *hlh-1* | II | 4518560 | 4522523 | 2.268 |
| *B0304.2* | II | 4523622 | 4524589 | 0.912 |
| *clec-140* | II | 4674420 | 4675729 | 1.134 |
| *T05A7.3* | II | 4675779 | 4676250 | 1.974 |
| *scc-1* | II | 4698721 | 4700994 | 2.087 |
| *rig-6* | II | 4798154 | 4812136 | 1.282 |
| *R05F9.6* | II | 4889437 | 4891427 | 0.896 |
| *btbd-10* | II | 4905008 | 4911835 | 1.202 |
| *ulp-2* | II | 4955646 | 4959876 | 1.112 |
| *F09E5.7* | II | 5352087 | 5353759 | 1.472 |
| *tofu-6* | II | 5421248 | 5422737 | 1.928 |
| *F56D1.14* | II | 5465988 | 5466135 | 1.728 |
| *F56D1.15* | II | 5466112 | 5466251 | 1.764 |
| *C17C3.15* | II | 5571074 | 5571969 | 1.372 |
| *cdc-14* | II | 5589186 | 5593628 | 0.911 |
| *C25H3.16* | II | 5666291 | 5666850 | 1.390 |
| *lec-5* | II | 5839639 | 5841134 | 1.389 |
| *nep-25* | II | 5842243 | 5844575 | 1.070 |
| *F59G1.10* | II | 5902211 | 5902355 | 2.014 |
| *lin-4* | II | 5902254 | 5902347 | 2.014 |
| *R12C12.6* | II | 6046286 | 6047867 | 1.581 |
| *F21H12.8* | II | 6083917 | 6084066 | 3.947 |
| *rgs-3* | II | 6100060 | 6110122 | 1.342 |
| *exp-1* | II | 6157167 | 6161094 | 1.621 |
| *upb-1* | II | 6288413 | 6290097 | 1.524 |
| *C29F5.1* | II | 6303424 | 6306283 | 1.971 |
| *C29F5.8* | II | 6306429 | 6308073 | 2.353 |
| *del-10* | II | 6474338 | 6482125 | 1.991 |
| *C56C10.6* | II | 6579702 | 6581336 | 2.088 |
| *kars-1* | II | 7070183 | 7072415 | 1.025 |
| *kat-1* | II | 7073796 | 7075551 | 1.243 |
| *glc-4* | II | 7172204 | 7174936 | 1.473 |
| *msra-1* | II | 7363216 | 7364107 | 1.778 |
| *R07G3.7* | II | 7610986 | 7613730 | 1.301 |
| *oig-4* | II | 7613541 | 7614434 | 0.881 |
| *cpna-5* | II | 7711871 | 7718591 | 2.141 |
| *B0495.15* | II | 7717241 | 7717382 | 2.077 |
| *cpna-2* | II | 7724903 | 7752715 | 1.358 |
| *glr-4* | II | 7785100 | 7794319 | 1.339 |
| *cex-2* | II | 7832844 | 7835414 | 1.503 |
| *spv-1* | II | 7912093 | 7928694 | 1.613 |
| *rpl-10* | II | 8143863 | 8144671 | 1.288 |
| *mig-5* | II | 8182323 | 8185023 | 0.828 |
| *cct-1* | II | 8185443 | 8187601 | 1.015 |
| *shw-1* | II | 8305745 | 8321471 | 1.409 |
| *T13H5.1* | II | 8487571 | 8509055 | 1.781 |
| *cht-2* | II | 8520905 | 8523490 | 0.801 |
| *F54C9.14* | II | 8562302 | 8563759 | 1.237 |
| *sgn-1* | II | 8782029 | 8783670 | 1.516 |
| *T14D7.4* | II | 8844702 | 8844924 | 2.426 |
| *T07D4.2* | II | 8869549 | 8874020 | 1.537 |
| *rpl-32* | II | 9056499 | 9057111 | 1.333 |
| *T24B8.4* | II | 9063687 | 9070904 | 1.120 |
| *aqp-2* | II | 9258913 | 9261994 | 1.438 |
| *C01G6.13* | II | 9262232 | 9262375 | 2.636 |
| *C01G6.2* | II | 9267336 | 9268303 | 1.483 |
| *tag-175* | II | 9323955 | 9324873 | 1.158 |
| *F42A8.4* | II | 9356152 | 9356295 | 1.186 |
| *C06C3.3* | II | 9369178 | 9372485 | 1.539 |
| *Y53C12B.1* | II | 9742982 | 9746074 | 1.022 |
| *pro-2* | II | 10339692 | 10345112 | 1.229 |
| *stip-1* | II | 10346981 | 10350007 | 1.750 |
| *ZK673.5* | II | 10461507 | 10461947 | 2.696 |
| *sdz-37* | II | 10464870 | 10465298 | 1.629 |
| *mnk-1* | II | 10542428 | 10555044 | 0.919 |
| *C14A4.13* | II | 10618471 | 10620503 | 1.187 |
| *glb-12* | II | 10729804 | 10731627 | 1.442 |
| *cdl-1* | II | 10785452 | 10787272 | 1.088 |
| *mix-1* | II | 10824294 | 10841424 | 1.588 |
| *M106.6* | II | 10840317 | 10840391 | 1.882 |
| *ptp-3* | II | 10968386 | 11012247 | 0.857 |
| *col-83* | II | 11044135 | 11045968 | 1.036 |
| *unc-53* | II | 11058096 | 11089456 | 2.329 |
| *C09H10.12* | II | 11081164 | 11081309 | 1.481 |
| *F37H8.5* | II | 11188206 | 11189437 | 2.937 |
| *T06D8.2* | II | 11226679 | 11232835 | 1.034 |
| *unc-130* | II | 11281986 | 11283953 | 1.485 |
| *C47G2.8* | II | 11294770 | 11296020 | 2.600 |
| *rsf-1* | II | 11306399 | 11311940 | 1.457 |
| *F54D5.7* | II | 11547481 | 11549313 | 0.971 |
| *F54D5.2* | II | 11573180 | 11575000 | 1.282 |
| *din-1* | II | 11614636 | 11633609 | 0.831 |
| *VF13D12L.3* | II | 11705989 | 11713610 | 1.109 |
| *F13D12.3* | II | 11720444 | 11722308 | 1.030 |
| *ldh-1* | II | 11724204 | 11726533 | 1.100 |
| *alh-8* | II | 11728949 | 11731226 | 1.453 |
| *F13D12.8* | II | 11731165 | 11732594 | 1.019 |
| *gpb-1* | II | 11742814 | 11748611 | 1.023 |
| *F43G6.10* | II | 11809347 | 11810307 | 0.918 |
| *K12D12.4* | II | 11863525 | 11865506 | 1.291 |
| *W03C9.6* | II | 11953239 | 11960743 | 1.077 |
| *F57G9.7* | II | 12394856 | 12397756 | 6.113 |
| *Y38E10A.8* | II | 12607200 | 12608788 | 1.631 |
| *Y46G5A.22* | II | 12816820 | 12818588 | 1.144 |
| *Y38F1A.1* | II | 12952490 | 12954832 | 1.776 |
| *Y38F1A.13* | II | 13020773 | 13021074 | 0.996 |
| *F42G4.2* | II | 13047941 | 13050807 | 0.829 |
| *zyx-1* | II | 13056814 | 13071530 | 1.287 |
| *E01G4.5* | II | 13472401 | 13473995 | 1.231 |
| *C01B9.6* | II | 13788897 | 13789046 | 0.857 |
| *Y39G8B.8* | II | 13993009 | 13993527 | 0.885 |
| *gur-4* | II | 14104773 | 14108072 | 1.322 |
| *Y48B6A.5* | II | 14172833 | 14175174 | 1.369 |
| *Y54G11A.11* | II | 14349783 | 14350795 | 1.206 |
| *C13B4.1* | II | 14470371 | 14479627 | 1.000 |
| *smg-9* | II | 14746890 | 14752489 | 2.907 |
| *pck-1* | III | 62888 | 66261 | 2.132 |
| *F40G9.17* | III | 194460 | 210063 | 1.281 |
| *Y55B1BR.6* | III | 453785 | 463811 | 1.843 |
| *helq-1* | III | 577684 | 587105 | 0.964 |
| *F42G9.10* | III | 766504 | 766653 | 1.997 |
| *F23H11.7* | III | 917088 | 919422 | 0.915 |
| *Y22D7AR.7* | III | 1683630 | 1688099 | 0.982 |
| *B0524.10* | III | 1918532 | 1918708 | 1.308 |
| *F53A3.7* | III | 1951526 | 1952032 | 0.789 |
| *mir-229* | III | 2172452 | 2172566 | 1.137 |
| *pbo-1* | III | 2681451 | 2686463 | 1.148 |
| *set-27* | III | 2700937 | 2707818 | 1.825 |
| *laf-1* | III | 2769358 | 2779053 | 1.523 |
| *H05C05.2* | III | 2962170 | 2965634 | 1.973 |
| *H05C05.3* | III | 2967048 | 2968932 | 1.503 |
| *phf-15* | III | 3322962 | 3331727 | 1.356 |
| *mlc-4* | III | 3392514 | 3393787 | 2.098 |
| *C32A3.7* | III | 3621520 | 3621644 | 0.869 |
| *unc-93* | III | 3644375 | 3648822 | 1.086 |
| *ccdc-47* | III | 3916933 | 3918809 | 1.478 |
| *tbb-2* | III | 4015769 | 4017643 | 0.922 |
| *H38K22.8* | III | 4317339 | 4317381 | 4.094 |
| *H38K22.7* | III | 4321748 | 4322867 | 1.120 |
| *B0285.16* | III | 4362461 | 4362677 | 1.740 |
| *rnp-4* | III | 4406286 | 4407326 | 1.982 |
| *sel-5* | III | 4571753 | 4578437 | 1.244 |
| *sel-2* | III | 4596134 | 4612384 | 1.144 |
| *acy-1* | III | 4722126 | 4730919 | 1.251 |
| *gipc-1* | III | 4870762 | 4872046 | 1.832 |
| *flh-2* | III | 4952124 | 4955171 | 0.981 |
| *C27F2.4* | III | 4958329 | 4960305 | 1.004 |
| *Y54H5A.2* | III | 5137252 | 5149578 | 0.859 |
| *K10D2.10* | III | 5180665 | 5180841 | 1.608 |
| *F52C9.5* | III | 5302218 | 5306324 | 0.844 |
| *ZK121.2* | III | 5341682 | 5344273 | 1.589 |
| *rpc-2* | III | 5557344 | 5564837 | 1.495 |
| *mlc-3* | III | 5565025 | 5567382 | 1.839 |
| *molo-1* | III | 5573994 | 5576684 | 1.374 |
| *mlp-1* | III | 5971530 | 5973664 | 1.499 |
| *anmt-2* | III | 6154844 | 6157758 | 1.153 |
| *F42A10.6* | III | 6176148 | 6176861 | 0.994 |
| *hmg-1.2* | III | 6280380 | 6282411 | 1.053 |
| *C56G2.9* | III | 6342481 | 6343287 | 2.102 |
| *C16A3.4* | III | 6381634 | 6383024 | 1.249 |
| *let-756* | III | 6407129 | 6410018 | 1.418 |
| *C05D11.5* | III | 6410033 | 6412014 | 1.194 |
| *dlc-1* | III | 6462907 | 6463801 | 0.990 |
| *pdfr-1* | III | 6632841 | 6652083 | 1.469 |
| *wdr-4* | III | 6725789 | 6728058 | 1.722 |
| *Y102E9.5* | III | 6728155 | 6730170 | 1.033 |
| *dig-1* | III | 6746308 | 6794764 | 1.352 |
| *T07E3.3* | III | 6903895 | 6905170 | 2.126 |
| *flap-1* | III | 6924097 | 6928817 | 0.855 |
| *C06E8.5* | III | 7002301 | 7005450 | 1.663 |
| *R151.14* | III | 7217055 | 7217110 | 2.037 |
| *C07H6.4* | III | 7499677 | 7503241 | 1.114 |
| *lig-4* | III | 7521004 | 7524836 | 1.407 |
| *lin-39* | III | 7528641 | 7536632 | 0.778 |
| *ZK783.6* | III | 7648788 | 7650005 | 1.949 |
| *egl-45* | III | 7824377 | 7828626 | 1.010 |
| *ZK688.9* | III | 7881113 | 7883003 | 0.992 |
| *F09G8.7* | III | 8267921 | 8268355 | 1.228 |
| *rpn-3* | III | 8447353 | 8449346 | 1.157 |
| *C06E1.1* | III | 8605832 | 8606622 | 1.573 |
| *F22B7.9* | III | 8652578 | 8653925 | 0.926 |
| *F22B7.t4* | III | 8653608 | 8653680 | 0.873 |
| *F22B7.15* | III | 8653608 | 8653681 | 0.873 |
| *snap-29* | III | 8776183 | 8777895 | 1.040 |
| *pat-2* | III | 8818899 | 8825270 | 1.431 |
| *ZK637.12* | III | 8922292 | 8925302 | 1.029 |
| *far-1* | III | 9080371 | 9081179 | 2.113 |
| *F02A9.9* | III | 9081466 | 9081595 | 2.278 |
| *far-2* | III | 9081679 | 9082511 | 1.684 |
| *emb-9* | III | 9336881 | 9344447 | 1.913 |
| *K04H4.2* | III | 9347714 | 9355480 | 1.034 |
| *spk-1* | III | 9456563 | 9463004 | 1.012 |
| *B0464.6* | III | 9462989 | 9465965 | 0.955 |
| *C15H7.9* | III | 9640123 | 9640341 | 1.486 |
| *ncx-7* | III | 9715883 | 9720291 | 1.733 |
| *cyld-1* | III | 9898602 | 9910658 | 0.969 |
| *K08E5.1* | III | 10141573 | 10146801 | 3.064 |
| *nac-3* | III | 10152795 | 10157923 | 1.581 |
| *Y70G10A.3* | III | 10238672 | 10244810 | 1.005 |
| *atx-2* | III | 10461389 | 10468051 | 0.819 |
| *unc-49* | III | 10520676 | 10532705 | 1.868 |
| *orc-4* | III | 10646170 | 10648861 | 0.940 |
| *cnt-2* | III | 10655018 | 10677521 | 1.001 |
| *sca-1* | III | 10811906 | 10816844 | 1.436 |
| *rsa-2* | III | 11063479 | 11068753 | 1.066 |
| *Y66D12A.9* | III | 11568137 | 11573343 | 1.631 |
| *immp-1* | III | 11774950 | 11776675 | 2.080 |
| *Y56A3A.18* | III | 11917836 | 11918719 | 1.201 |
| *Y56A3A.7* | III | 11934002 | 11947017 | 1.470 |
| *mir-86* | III | 11936637 | 11936734 | 1.680 |
| *zip-1* | III | 12333745 | 12345347 | 0.949 |
| *Y111B2A.3* | III | 12496538 | 12499352 | 0.843 |
| *T05D4.2* | III | 13561729 | 13563082 | 1.465 |
| *aldo-1* | III | 13563346 | 13564990 | 1.622 |
| *T12D8.10* | III | 13641795 | 13642327 | 0.905 |
| *daf-18* | IV | 419938 | 425181 | 1.775 |
| *prmt-4* | IV | 602991 | 603643 | 1.360 |
| *kvs-5* | IV | 736675 | 753316 | 1.523 |
| *C44B12.9* | IV | 1103541 | 1104609 | 1.733 |
| *ost-1* | IV | 1107396 | 1110856 | 1.921 |
| *K08D12.3* | IV | 1710265 | 1711127 | 1.181 |
| *Y41D4A.7* | IV | 1752270 | 1761647 | 2.007 |
| *Y41D4A.3* | IV | 1753824 | 1755750 | 1.761 |
| *Y48A5A.1* | IV | 1965481 | 1969650 | 3.255 |
| *Y76B12C.4* | IV | 1970734 | 1972977 | 2.909 |
| *R05C11.2* | IV | 2057878 | 2059111 | 1.927 |
| *test-1* | IV | 2136895 | 2146461 | 1.376 |
| *Y69A2AR.19* | IV | 2488820 | 2494135 | 0.775 |
| *Y69A2AR.41* | IV | 2639797 | 2639880 | 0.785 |
| *Y69A2AR.t2* | IV | 2639809 | 2639880 | 0.785 |
| *Y69A2AR.37* | IV | 2664753 | 2665339 | 0.910 |
| *Y54G2A.73* | IV | 3000541 | 3008892 | 1.089 |
| *T23E1.6* | IV | 3168800 | 3168949 | 2.689 |
| *CC8.2* | IV | 3645166 | 3648684 | 1.301 |
| *nape-2* | IV | 3735845 | 3738917 | 0.801 |
| *vab-2* | IV | 3761192 | 3766826 | 1.116 |
| *bris-1* | IV | 3807870 | 3819328 | 1.201 |
| *zag-1* | IV | 3855843 | 3861552 | 1.190 |
| *aat-8* | IV | 3862129 | 3865042 | 2.112 |
| *R08C7.8* | IV | 4433049 | 4434541 | 1.266 |
| *fbxb-74* | IV | 4437786 | 4439290 | 1.558 |
| *M01H9.3* | IV | 4468505 | 4477059 | 1.289 |
| *gbb-2* | IV | 4489012 | 4506708 | 1.181 |
| *R105.4* | IV | 4635451 | 4635961 | 1.751 |
| *R105.5* | IV | 4636301 | 4636970 | 4.100 |
| *F29B9.5* | IV | 4652459 | 4653159 | 1.117 |
| *jmjd-1.2* | IV | 4660991 | 4665204 | 1.011 |
| *F29B9.1* | IV | 4665251 | 4667293 | 1.543 |
| *bus-4* | IV | 4688269 | 4691936 | 1.041 |
| *timm-17B.1* | IV | 4744377 | 4745492 | 1.618 |
| *eif-1.A* | IV | 4810934 | 4811983 | 1.416 |
| *str-170* | IV | 4877402 | 4880315 | 1.598 |
| *ivd-1* | IV | 5086839 | 5088883 | 1.472 |
| *F36H12.4* | IV | 5269090 | 5269626 | 1.764 |
| *ZK616.61* | IV | 5405645 | 5407375 | 2.420 |
| *Y4C6A.4* | IV | 5452967 | 5453640 | 1.269 |
| *nhr-46* | IV | 5747376 | 5757862 | 1.053 |
| *B0350.79* | IV | 5980619 | 5980754 | 1.914 |
| *21ur-14209* | IV | 5981091 | 5981111 | 1.751 |
| *C46G7.5* | IV | 6010352 | 6011645 | 4.726 |
| *pqn-22* | IV | 6019705 | 6033639 | 1.698 |
| *M03D4.74* | IV | 6140884 | 6141064 | 1.124 |
| *ugt-24* | IV | 6206843 | 6209527 | 1.196 |
| *T05A12.3* | IV | 6883576 | 6887662 | 1.621 |
| *ZC477.4* | IV | 7098542 | 7098784 | 1.396 |
| *ZC477.16* | IV | 7098571 | 7098933 | 1.396 |
| *unc-82* | IV | 7424498 | 7444660 | 1.413 |
| *B0496.19* | IV | 7428467 | 7428881 | 1.376 |
| *B0496.1* | IV | 7446390 | 7447466 | 1.342 |
| *mrpl-1* | IV | 7707575 | 7709084 | 1.586 |
| *C06A6.14* | IV | 7845656 | 7845868 | 1.145 |
| *C49H3.6* | IV | 7903740 | 7907083 | 1.815 |
| *gcy-8* | IV | 7928159 | 7933775 | 0.911 |
| *sax-7* | IV | 8062669 | 8084087 | 1.245 |
| *anr-44* | IV | 8231731 | 8235014 | 1.281 |
| *K07H8.5* | IV | 8271054 | 8272524 | 1.843 |
| *K07H8.9* | IV | 8285175 | 8286566 | 1.647 |
| *M116.2* | IV | 8398923 | 8399762 | 1.819 |
| *F42A9.18* | IV | 8605512 | 8607840 | 2.952 |
| *F42A9.11* | IV | 8627644 | 8627839 | 1.031 |
| *ipla-5* | IV | 8715247 | 8717881 | 1.393 |
| *alp-1* | IV | 8852500 | 8862228 | 1.039 |
| *srx-15* | IV | 9021515 | 9023607 | 1.966 |
| *mans-4* | IV | 9076501 | 9078807 | 1.688 |
| *hsp-12.6* | IV | 9446385 | 9446884 | 2.169 |
| *elo-1* | IV | 9526359 | 9528350 | 1.034 |
| *F56H11.9* | IV | 9538440 | 9538571 | 1.561 |
| *T13H10.9* | IV | 9947771 | 9947981 | 3.171 |
| *dhp-2* | IV | 9978122 | 9980770 | 1.838 |
| *frk-1* | IV | 10043183 | 10045739 | 1.989 |
| *ttbk-4* | IV | 10073404 | 10075166 | 1.336 |
| *ttr-54* | IV | 10155775 | 10156771 | 2.699 |
| *C43F9.5* | IV | 10589294 | 10590749 | 2.428 |
| *fmo-1* | IV | 10661857 | 10664404 | 1.814 |
| *K08C7.7* | IV | 10664412 | 10665685 | 2.815 |
| *epi-1* | IV | 10666827 | 10679289 | 1.500 |
| *fmo-2* | IV | 10682524 | 10684385 | 1.274 |
| *R102.2* | IV | 10687592 | 10688135 | 1.207 |
| *allo-1* | IV | 10695309 | 10697071 | 1.455 |
| *T11G6.2* | IV | 10855485 | 10858559 | 0.966 |
| *Y43E12A.3* | IV | 10987906 | 10989182 | 1.037 |
| *F01G4.6* | IV | 11146940 | 11149504 | 0.885 |
| *fbxb-76* | IV | 11378174 | 11379348 | 1.915 |
| *H21P03.6* | IV | 11494936 | 11495338 | 0.876 |
| *H21P03.7* | IV | 11494936 | 11495254 | 0.876 |
| *F12F6.19* | IV | 11572528 | 11572671 | 1.383 |
| *T12A7.12* | IV | 11748939 | 11749074 | 1.822 |
| *T12A7.6* | IV | 11749154 | 11751074 | 1.787 |
| *scl-18* | IV | 11752987 | 11754387 | 1.058 |
| *T12A7.9* | IV | 11755274 | 11755970 | 1.909 |
| *T12A7.2* | IV | 11756381 | 11761064 | 0.991 |
| *M117.1* | IV | 11814247 | 11819612 | 1.079 |
| *ZK829.9* | IV | 11970534 | 11972789 | 1.451 |
| *unc-22* | IV | 11972697 | 12011007 | 2.027 |
| *ZK617.21* | IV | 11992606 | 11992746 | 1.387 |
| *ZK617.23* | IV | 12010399 | 12010681 | 2.105 |
| *ZK617.8* | IV | 12012388 | 12012667 | 1.881 |
| *ZK617.18* | IV | 12012463 | 12012876 | 1.881 |
| *col-129* | IV | 12108672 | 12110275 | 1.162 |
| *dhhc-6* | IV | 12124952 | 12126785 | 0.991 |
| *marb-1* | IV | 12208192 | 12209010 | 1.363 |
| *ccr-4* | IV | 12361542 | 12371852 | 1.088 |
| *spe-44* | IV | 12452066 | 12454215 | 1.742 |
| *ark-1* | IV | 12622093 | 12631022 | 0.796 |
| *nas-14* | IV | 13170544 | 13173694 | 2.217 |
| *lev-1* | IV | 13174157 | 13179457 | 1.530 |
| *W02A2.11* | IV | 13333115 | 13333300 | 1.536 |
| *Y45F10B.13* | IV | 13549605 | 13561729 | 1.571 |
| *qui-1* | IV | 13561861 | 13574440 | 0.869 |
| *tlp-1* | IV | 13701240 | 13704082 | 1.691 |
| *lin-66* | IV | 13888089 | 13893985 | 0.797 |
| *F02H6.1* | IV | 14199263 | 14201780 | 1.344 |
| *21ur-5470* | IV | 14224943 | 14224963 | 1.295 |
| *Y57G11C.8* | IV | 14781837 | 14782731 | 3.020 |
| *Y41E3.6* | IV | 15006247 | 15007193 | 1.712 |
| *Y41E3.5* | IV | 15007803 | 15008173 | 2.089 |
| *Y40H7A.15* | IV | 15194534 | 15195852 | 2.589 |
| *ccg-1* | IV | 15270160 | 15275835 | 0.955 |
| *Y105C5A.14* | IV | 15646970 | 15648298 | 1.327 |
| *Y105C5A.1273* | IV | 15791146 | 15791343 | 0.988 |
| *Y105C5B.9* | IV | 15930581 | 15931813 | 1.187 |
| *Y65A5A.1* | IV | 16398839 | 16399875 | 1.232 |
| *Y116A8C.20* | IV | 17040913 | 17041523 | 2.091 |
| *Y116A8C.43* | IV | 17088851 | 17090646 | 1.108 |
| *itsn-1* | IV | 17119684 | 17126266 | 0.807 |
| *F11E6.7* | IV | 17457834 | 17461272 | 0.836 |
| *gba-3* | IV | 17473334 | 17479397 | 2.601 |
| *npr-5* | V | 276989 | 281716 | 1.963 |
| *nhr-58* | V | 532290 | 534064 | 0.902 |
| *C14C6.12* | V | 570571 | 571205 | 2.137 |
| *icl-1* | V | 724207 | 728103 | 1.253 |
| *srh-2* | V | 728312 | 734279 | 0.790 |
| *cmd-1* | V | 1156271 | 1158174 | 1.056 |
| *madd-2* | V | 1223901 | 1235445 | 1.343 |
| *Y75B7B.1* | V | 1270911 | 1273043 | 1.181 |
| *hsp-16.2* | V | 1804334 | 1804971 | 2.897 |
| *srp-3* | V | 1908855 | 1910692 | 1.509 |
| *bgnt-1.2* | V | 2115490 | 2118644 | 1.165 |
| *ketn-1* | V | 2781943 | 2807934 | 1.278 |
| *F54E2.2* | V | 2796298 | 2797010 | 1.315 |
| *F54E2.8* | V | 2802073 | 2802874 | 2.177 |
| *srab-11* | V | 3145203 | 3147032 | 2.919 |
| *T28A11.3* | V | 3275333 | 3276110 | 1.207 |
| *T28A11.2* | V | 3280379 | 3281169 | 2.563 |
| *F35F10.5* | V | 3284929 | 3285792 | 1.968 |
| *F35F10.6* | V | 3287995 | 3289385 | 2.578 |
| *F35F10.4* | V | 3289635 | 3293435 | 2.173 |
| *arrd-18* | V | 3310123 | 3312117 | 1.584 |
| *arrd-19* | V | 3313286 | 3315516 | 0.910 |
| *C17B7.4* | V | 3339348 | 3340092 | 1.880 |
| *C17B7.3* | V | 3342804 | 3343865 | 1.281 |
| *arrd-21* | V | 3389330 | 3390952 | 1.365 |
| *T20D4.17* | V | 3395275 | 3395966 | 3.721 |
| *T20D4.11* | V | 3398690 | 3399640 | 1.772 |
| *T20D4.20* | V | 3401645 | 3402356 | 1.914 |
| *T20D4.9* | V | 3402631 | 3405161 | 1.425 |
| *srw-130* | V | 3546969 | 3549685 | 1.511 |
| *acr-23* | V | 3651686 | 3659499 | 1.378 |
| *str-113* | V | 3664871 | 3666987 | 1.577 |
| *Y45G5AM.3* | V | 4164554 | 4166825 | 1.056 |
| *F13A2.14* | V | 4379643 | 4381592 | 2.179 |
| *unc-62* | V | 4497463 | 4511449 | 0.909 |
| *sos-1* | V | 4517643 | 4549291 | 0.844 |
| *prx-6* | V | 4715388 | 4720148 | 1.234 |
| *C18G1.1* | V | 4777960 | 4780019 | 1.748 |
| *acl-6* | V | 5433392 | 5437066 | 1.073 |
| *F25B4.4* | V | 5684875 | 5685728 | 2.164 |
| *F44E7.15* | V | 5780500 | 5780645 | 0.869 |
| *mir-253* | V | 5780510 | 5780616 | 0.869 |
| *F26F12.19* | V | 5851249 | 5851398 | 1.641 |
| *dhs-17* | V | 6026330 | 6034923 | 1.547 |
| *ttn-1* | V | 6120909 | 6202626 | 1.057 |
| *W06H8.11* | V | 6202753 | 6202918 | 1.686 |
| *W06H8.4* | V | 6204875 | 6206134 | 1.710 |
| *K08B12.4* | V | 6235913 | 6236854 | 2.126 |
| *sago-1* | V | 6303771 | 6308337 | 0.979 |
| *T28C12.8* | V | 6342645 | 6342823 | 1.977 |
| *acl-9* | V | 6453196 | 6454973 | 2.464 |
| *EGAP9.4* | V | 6576867 | 6577541 | 1.674 |
| *T25F10.9* | V | 6757042 | 6757176 | 1.755 |
| *dbl-1* | V | 6758135 | 6760418 | 1.571 |
| *bbs-8* | V | 6762476 | 6765072 | 1.970 |
| *clik-1* | V | 6765560 | 6768732 | 1.316 |
| *T25F10.1* | V | 6768429 | 6768788 | 1.679 |
| *srh-30* | V | 6769662 | 6771704 | 1.970 |
| *T19F4.6* | V | 6879479 | 6879874 | 1.476 |
| *tofu-2* | V | 7049434 | 7051311 | 1.312 |
| *F09G2.12* | V | 7171103 | 7171252 | 1.644 |
| *F09G2.13* | V | 7171167 | 7171391 | 1.615 |
| *F09G2.10* | V | 7175054 | 7175254 | 2.442 |
| *F09G2.8* | V | 7188332 | 7193662 | 0.845 |
| *C13A2.6* | V | 7269731 | 7272787 | 1.793 |
| *F10G2.10* | V | 7322215 | 7323790 | 1.805 |
| *F10G2.2* | V | 7331168 | 7331779 | 1.879 |
| *C03G6.5* | V | 7365251 | 7366204 | 1.866 |
| *srbc-11* | V | 7493880 | 7495289 | 1.704 |
| *srd-30* | V | 7629804 | 7631298 | 1.098 |
| *F40A3.2* | V | 7878914 | 7881403 | 1.401 |
| *cri-2* | V | 8201808 | 8202546 | 1.331 |
| *ZC190.4* | V | 8666065 | 8671680 | 1.194 |
| *K06C4.18* | V | 8920198 | 8920347 | 1.384 |
| *K06C4.22* | V | 8920402 | 8920582 | 2.145 |
| *fip-2* | V | 9067976 | 9068390 | 3.681 |
| *F21C10.15* | V | 9112133 | 9112271 | 1.556 |
| *F21C10.7* | V | 9112767 | 9122180 | 1.255 |
| *glo-4* | V | 9234610 | 9243221 | 1.621 |
| *F44A2.5* | V | 9297632 | 9302158 | 0.997 |
| *gsnl-1* | V | 9497042 | 9499372 | 1.192 |
| *mdf-1* | V | 9541782 | 9544226 | 1.360 |
| *anmt-3* | V | 9956879 | 9958331 | 1.375 |
| *ruvb-1* | V | 9978791 | 9980802 | 1.277 |
| *F57A8.1* | V | 10037340 | 10048726 | 1.103 |
| *R04B5.5* | V | 10087480 | 10088998 | 0.963 |
| *C51E3.9* | V | 10145905 | 10148977 | 1.309 |
| *ZK856.7* | V | 10201856 | 10202610 | 0.926 |
| *fubl-1* | V | 10236160 | 10238902 | 0.817 |
| *B0024.11* | V | 10317490 | 10319534 | 0.999 |
| *F22E12.1* | V | 10453748 | 10457347 | 1.574 |
| *chw-1* | V | 10462282 | 10464660 | 1.615 |
| *T28B11.1* | V | 10712165 | 10732507 | 0.889 |
| *F32D8.13* | V | 10895654 | 10896543 | 1.029 |
| *F32D8.7* | V | 10898435 | 10900701 | 1.184 |
| *col-153* | V | 10949857 | 10951821 | 1.292 |
| *C13G3.5* | V | 11031334 | 11031480 | 0.819 |
| *erfa-3* | V | 11110063 | 11113103 | 1.447 |
| *lgc-49* | V | 11191283 | 11193638 | 2.705 |
| *C03E10.8* | V | 11281583 | 11281725 | 1.983 |
| *C03E10.9* | V | 11281951 | 11282097 | 1.690 |
| *T03F7.11* | V | 11307918 | 11308060 | 2.112 |
| *F47G9.4* | V | 11317214 | 11321786 | 1.442 |
| *C55A6.10* | V | 11505352 | 11508271 | 1.536 |
| *ttll-5* | V | 11509068 | 11512318 | 1.500 |
| *C55A6.3* | V | 11512313 | 11513436 | 1.403 |
| *C55A6.6* | V | 11513665 | 11514737 | 1.290 |
| *C55A6.11* | V | 11520515 | 11521554 | 2.552 |
| *F46F3.6* | V | 11675086 | 11675304 | 1.442 |
| *F46F3.15* | V | 11675149 | 11675358 | 1.442 |
| *F46F3.21* | V | 11679713 | 11679920 | 2.031 |
| *mir-250* | V | 11769917 | 11770015 | 1.543 |
| *K12G11.14* | V | 11882456 | 11882750 | 2.840 |
| *R31.2* | V | 11917564 | 11924654 | 0.836 |
| *R31.5* | V | 11919733 | 11919945 | 2.493 |
| *vab-8* | V | 12213327 | 12225935 | 1.011 |
| *myo-3* | V | 12226816 | 12234343 | 1.636 |
| *T04H1.2* | V | 12241962 | 12245059 | 0.802 |
| *F23H12.18* | V | 12340807 | 12340936 | 1.497 |
| *F23H12.10* | V | 12373663 | 12374231 | 1.407 |
| *F45D3.2* | V | 12541040 | 12542973 | 1.087 |
| *F45D3.3* | V | 12549216 | 12550385 | 1.767 |
| *F45D3.4* | V | 12552211 | 12553301 | 1.128 |
| *C14C10.5* | V | 12600192 | 12607304 | 0.832 |
| *ttbk-7* | V | 12891523 | 12895872 | 1.360 |
| *eif-3.K* | V | 12954002 | 12955229 | 2.439 |
| *R186.3* | V | 12966000 | 12967210 | 0.957 |
| *nhr-129* | V | 13329193 | 13331019 | 2.057 |
| *somi-1* | V | 13386065 | 13391393 | 1.482 |
| *F53F1.3* | V | 13408242 | 13409832 | 3.313 |
| *tbcb-1* | V | 13592124 | 13593183 | 0.802 |
| *D1086.3* | V | 14092792 | 14093986 | 1.718 |
| *D1086.2* | V | 14097038 | 14098668 | 3.795 |
| *D1086.12* | V | 14101186 | 14102821 | 2.212 |
| *H39E23.2* | V | 14110361 | 14112027 | 1.452 |
| *F40G12.3* | V | 14261990 | 14267317 | 2.313 |
| *Y49A3A.3* | V | 14354410 | 14355754 | 1.099 |
| *gar-3* | V | 14564752 | 14579719 | 1.303 |
| *pmp-3* | V | 14646739 | 14653464 | 1.592 |
| *hsp-90* | V | 14684918 | 14688543 | 0.841 |
| *unc-112* | V | 14691854 | 14697107 | 1.445 |
| *W06A7.4* | V | 14824664 | 14829533 | 1.489 |
| *ZC412.3* | V | 14868645 | 14871547 | 1.223 |
| *fib-1* | V | 15000653 | 15002273 | 0.962 |
| *C25D7.15* | V | 15060249 | 15061530 | 1.574 |
| *unc-76* | V | 15071748 | 15080152 | 1.106 |
| *Y75B12B.3* | V | 15185891 | 15186300 | 2.729 |
| *linc-108* | V | 15565170 | 15566425 | 1.135 |
| *acs-2* | V | 15567394 | 15569889 | 2.097 |
| *T03E6.9* | V | 16598989 | 16600723 | 1.764 |
| *phy-3* | V | 16833316 | 16839141 | 1.695 |
| *ZK218.5* | V | 17101132 | 17102017 | 3.740 |
| *T27C5.16* | V | 17407974 | 17408383 | 2.591 |
| *twk-42* | V | 17726799 | 17730381 | 2.077 |
| *ttr-25* | V | 18556714 | 18558099 | 1.217 |
| *Y17D7C.3* | V | 18720589 | 18723771 | 2.152 |
| *F11D11.16* | V | 18756846 | 18757678 | 3.351 |
| *Y39B6A.8* | V | 19140795 | 19145542 | 0.960 |
| *C43D7.10* | V | 19308707 | 19309687 | 1.546 |
| *Y43F8C.7* | V | 19641292 | 19644083 | 0.867 |
| *fbxa-131* | V | 19738417 | 19739393 | 2.038 |
| *Y113G7A.13* | V | 20148195 | 20150357 | 1.780 |
| *srxa-6* | V | 20734983 | 20736149 | 2.510 |
| *clec-264* | V | 20847702 | 20850666 | 1.423 |
| *Y73B3A.1* | X | 42728 | 46924 | 1.247 |
| *Y73B3A.t1* | X | 86887 | 86958 | 4.164 |
| *unc-96* | X | 611120 | 621290 | 1.245 |
| *mam-1* | X | 882967 | 888394 | 1.231 |
| *rme-6* | X | 1097152 | 1103320 | 1.110 |
| *ife-4* | X | 1104212 | 1106247 | 1.503 |
| *C05D9.7* | X | 1117192 | 1119405 | 1.248 |
| *tmc-1* | X | 1163153 | 1177172 | 1.216 |
| *hot-8* | X | 1180284 | 1187932 | 1.378 |
| *dgn-2* | X | 1365474 | 1367516 | 1.789 |
| *F56C3.10* | X | 1381375 | 1381665 | 1.584 |
| *F07G6.3* | X | 1723092 | 1724888 | 2.363 |
| *fbxa-40* | X | 1937947 | 1942199 | 1.090 |
| *txdc-12.2* | X | 2074448 | 2075944 | 1.694 |
| *K02E10.7* | X | 2501003 | 2508549 | 1.387 |
| *R11B5.10* | X | 2518676 | 2518739 | 1.516 |
| *F52H2.9* | X | 2552309 | 2552440 | 1.543 |
| *F54G2.3* | X | 2618448 | 2618652 | 1.443 |
| *tra-4* | X | 2853678 | 2857603 | 0.933 |
| *pfk-1.1* | X | 2940872 | 2945481 | 1.394 |
| *Y41G9A.10* | X | 2945803 | 2946570 | 1.774 |
| *osm-5* | X | 2984278 | 2991375 | 1.711 |
| *M02F4.11* | X | 3024214 | 3024437 | 2.834 |
| *aqp-7* | X | 3025505 | 3026976 | 2.479 |
| *F56E3.10* | X | 3183345 | 3183545 | 1.567 |
| *tba-9* | X | 3252963 | 3256265 | 2.552 |
| *lbp-2* | X | 3260610 | 3261470 | 2.587 |
| *F11D5.32* | X | 3313067 | 3313216 | 2.102 |
| *acp-1* | X | 3382868 | 3385547 | 2.018 |
| *C04F6.7* | X | 3416888 | 3419213 | 1.130 |
| *npr-16* | X | 3528812 | 3531826 | 1.576 |
| *gyg-1* | X | 3537641 | 3540940 | 1.114 |
| *sax-1* | X | 3646320 | 3650150 | 1.930 |
| *tsp-17* | X | 3681170 | 3684232 | 1.617 |
| *tag-278* | X | 3684212 | 3689205 | 1.050 |
| *C02F12.8* | X | 3690143 | 3693238 | 0.881 |
| *C01C4.7* | X | 3701768 | 3701819 | 1.494 |
| *C01C4.3* | X | 3706551 | 3713382 | 1.577 |
| *ceh-18* | X | 3843367 | 3857797 | 0.918 |
| *sup-12* | X | 3904591 | 3907671 | 2.012 |
| *T22B2.5* | X | 3908144 | 3911380 | 3.353 |
| *T22B2.6* | X | 3914765 | 3915848 | 2.746 |
| *adt-3* | X | 3931631 | 3937897 | 1.016 |
| *ZK470.4* | X | 4139516 | 4139767 | 1.344 |
| *C24A8.5* | X | 4328400 | 4328916 | 1.587 |
| *F14H12.8* | X | 4363607 | 4365069 | 0.886 |
| *lst-2* | X | 4378004 | 4383366 | 1.032 |
| *mltn-13* | X | 4442152 | 4448383 | 1.410 |
| *F46H6.8* | X | 4498491 | 4498652 | 1.570 |
| *pdi-2* | X | 4524155 | 4526536 | 0.801 |
| *pqn-65* | X | 4610926 | 4620423 | 0.913 |
| *mig-13* | X | 4796095 | 4800128 | 1.387 |
| *T07F12.9* | X | 4877264 | 4877409 | 1.308 |
| *M03F4.4* | X | 4957329 | 4958067 | 1.275 |
| *act-4* | X | 4960553 | 4964416 | 1.344 |
| *T03G11.14* | X | 5172931 | 5173261 | 2.409 |
| *T03G11.19* | X | 5193554 | 5193755 | 1.250 |
| *pqn-62* | X | 5196110 | 5204250 | 1.171 |
| *T03G11.12* | X | 5199890 | 5199952 | 1.540 |
| *H28G03.2* | X | 5205760 | 5216230 | 0.889 |
| *H28G03.1* | X | 5216363 | 5219095 | 1.182 |
| *C41A3.2* | X | 5406562 | 5414350 | 1.299 |
| *mec-2* | X | 5575932 | 5590815 | 1.325 |
| *unc-97* | X | 5593133 | 5594965 | 0.988 |
| *F13D11.17* | X | 5819105 | 5819343 | 2.308 |
| *hbl-1* | X | 5822277 | 5827755 | 1.123 |
| *puf-9* | X | 5841592 | 5845987 | 0.812 |
| *acs-17* | X | 5937506 | 5944510 | 0.872 |
| *R07E4.5* | X | 5949529 | 5953174 | 1.425 |
| *kin-2* | X | 5970677 | 5977854 | 0.907 |
| *bca-1* | X | 6201875 | 6204922 | 1.485 |
| *lrch-1* | X | 6208761 | 6213911 | 1.803 |
| *C14F11.17* | X | 6224890 | 6225159 | 1.969 |
| *C14F11.32* | X | 6224890 | 6225090 | 1.969 |
| *lev-9* | X | 6281847 | 6291732 | 1.545 |
| *T07H6.7* | X | 6291493 | 6291695 | 1.583 |
| *T07H6.1* | X | 6300138 | 6305211 | 0.868 |
| *T22E5.12* | X | 6402683 | 6402881 | 2.034 |
| *T22E5.8* | X | 6402763 | 6403012 | 1.787 |
| *mup-2* | X | 6408470 | 6410359 | 1.610 |
| *T22E5.10* | X | 6410539 | 6410672 | 1.688 |
| *T22E5.6* | X | 6414148 | 6416400 | 1.121 |
| *glr-8* | X | 6506884 | 6509882 | 2.032 |
| *ttr-6* | X | 6596425 | 6597121 | 1.531 |
| *K11G12.6* | X | 6725781 | 6729748 | 1.217 |
| *H22K11.7* | X | 6777930 | 6778153 | 2.383 |
| *atg-2* | X | 6809104 | 6817266 | 0.804 |
| *F41B4.1* | X | 6823890 | 6825862 | 1.119 |
| *F41B4.2* | X | 6831251 | 6835266 | 1.529 |
| *C16E9.1* | X | 6940253 | 6943713 | 1.476 |
| *C16E9.15* | X | 6961303 | 6961673 | 2.174 |
| *lam-2* | X | 7143918 | 7151235 | 1.254 |
| *C54D1.9* | X | 7151458 | 7151745 | 1.639 |
| *pgp-10* | X | 7159036 | 7166021 | 1.351 |
| *bar-1* | X | 7166005 | 7170625 | 1.588 |
| *syd-9* | X | 7218109 | 7226825 | 1.408 |
| *F46H5.3* | X | 7230330 | 7238662 | 0.812 |
| *ttr-59* | X | 7329818 | 7330669 | 1.199 |
| *F46C8.3* | X | 7525571 | 7527433 | 1.524 |
| *C44E12.4* | X | 7606460 | 7606664 | 1.715 |
| *F26A10.20* | X | 7641673 | 7641783 | 1.861 |
| *stn-2* | X | 7676511 | 7680629 | 0.953 |
| *kvs-1* | X | 7690590 | 7707086 | 1.466 |
| *C14F5.7* | X | 7945252 | 7945391 | 2.109 |
| *C14F5.8* | X | 7948572 | 7948798 | 1.995 |
| *dct-1* | X | 7954355 | 7956211 | 1.641 |
| *dim-1* | X | 8050476 | 8058220 | 2.449 |
| *tnt-2* | X | 8721557 | 8723552 | 1.658 |
| *EGAP4.1* | X | 8726798 | 8727406 | 2.369 |
| *zig-12* | X | 8763907 | 8766280 | 1.425 |
| *unc-27* | X | 8789456 | 8790621 | 1.918 |
| *vav-1* | X | 9216867 | 9232075 | 1.031 |
| *lgc-4* | X | 9235103 | 9242223 | 1.004 |
| *tmc-2* | X | 9303724 | 9310439 | 1.533 |
| *rrc-1* | X | 9823752 | 9831249 | 0.913 |
| *R07B1.23* | X | 9842186 | 9842329 | 1.921 |
| *R07B1.16* | X | 9842397 | 9842525 | 1.350 |
| *tsp-21* | X | 9932371 | 9937133 | 1.631 |
| *F19C6.3* | X | 10005733 | 10008755 | 2.345 |
| *unc-58* | X | 10104612 | 10125483 | 1.644 |
| *T06H11.t1* | X | 10124498 | 10124579 | 1.463 |
| *erd-2.1* | X | 10156858 | 10160438 | 0.857 |
| *sex-1* | X | 10201329 | 10205433 | 1.595 |
| *ZC504.11* | X | 10410819 | 10410890 | 2.796 |
| *acr-8* | X | 10412725 | 10416630 | 1.200 |
| *syd-2* | X | 10549123 | 10555239 | 1.044 |
| *sdn-1* | X | 10589780 | 10593334 | 1.446 |
| *F47B10.8* | X | 10905181 | 10910768 | 1.710 |
| *dgn-1* | X | 10920533 | 10928998 | 1.435 |
| *T21B6.17* | X | 10937569 | 10937909 | 3.819 |
| *pho-7* | X | 10938197 | 10941355 | 2.041 |
| *T21B6.3* | X | 10945160 | 10948970 | 2.043 |
| *W04G3.12* | X | 11076182 | 11076767 | 1.987 |
| *F38B2.4* | X | 11286443 | 11287717 | 1.761 |
| *inx-2* | X | 11294962 | 11296731 | 0.992 |
| *F08G12.3* | X | 11300283 | 11302094 | 0.958 |
| *oct-2* | X | 11346455 | 11350244 | 0.928 |
| *F42E11.6* | X | 11369047 | 11369158 | 3.414 |
| *T25C12.10* | X | 11464663 | 11464786 | 1.305 |
| *lin-14* | X | 11465557 | 11484368 | 1.070 |
| *C35C5.10* | X | 11554795 | 11559858 | 0.931 |
| *C35C5.15* | X | 11560203 | 11560460 | 2.131 |
| *C35C5.17* | X | 11560220 | 11560467 | 2.131 |
| *abts-2* | X | 11585675 | 11589757 | 1.289 |
| *F52D10.2* | X | 11589686 | 11591719 | 1.812 |
| *W03G11.12* | X | 12085058 | 12085204 | 0.795 |
| *col-181* | X | 12104930 | 12106081 | 1.220 |
| *E01G6.7* | X | 12252489 | 12252749 | 1.395 |
| *lin-2* | X | 12399517 | 12414037 | 0.856 |
| *T18D3.7* | X | 12434154 | 12443915 | 2.080 |
| *str-123* | X | 12790336 | 12791751 | 1.182 |
| *K02A4.8* | X | 12878090 | 12878222 | 0.947 |
| *nlp-3* | X | 13177856 | 13178447 | 2.756 |
| *asp-4* | X | 13218977 | 13221462 | 1.282 |
| *skr-20* | X | 13222920 | 13223546 | 2.418 |
| *F02C12.1* | X | 13402083 | 13406415 | 1.380 |
| *F02D10.6* | X | 13449586 | 13451678 | 0.851 |
| *sad-1* | X | 13487555 | 13500662 | 1.377 |
| *cah-4* | X | 13542574 | 13544599 | 1.414 |
| *M03B6.1* | X | 13841610 | 13843108 | 1.651 |
| *hlb-1* | X | 13865038 | 13882693 | 1.242 |
| *irld-24* | X | 13986607 | 13989009 | 1.692 |
| *C04C11.19* | X | 14039816 | 14040019 | 2.612 |
| *C33G3.10* | X | 14054048 | 14054383 | 1.832 |
| *C33G3.11* | X | 14054785 | 14054909 | 1.430 |
| *madd-3* | X | 14284775 | 14293993 | 1.491 |
| *prx-1* | X | 14313721 | 14326800 | 1.009 |
| *his-24* | X | 14490094 | 14491115 | 0.954 |
| *M163.16* | X | 14490327 | 14490548 | 0.831 |
| *C26G2.4* | X | 14653660 | 14653883 | 1.716 |
| *C26G2.5* | X | 14653750 | 14653883 | 1.716 |
| *syg-2* | X | 14654972 | 14662841 | 1.981 |
| *F40E10.14* | X | 14684549 | 14684644 | 1.802 |
| *C05G5.1* | X | 14734443 | 14740088 | 1.383 |
| *mbk-1* | X | 14819909 | 14824540 | 1.251 |
| *rgs-10* | X | 14962922 | 14963995 | 0.931 |
| *rbg-1* | X | 14983533 | 14987365 | 1.988 |
| *T10B10.12* | X | 15189335 | 15189664 | 1.083 |
| *T10B10.11* | X | 15189419 | 15189680 | 1.083 |
| *dyn-1* | X | 15568890 | 15573693 | 0.911 |
| *C02C6.8* | X | 15575526 | 15575801 | 2.184 |
| *C02C6.6* | X | 15575555 | 15575757 | 2.184 |
| *K09A9.11* | X | 15580510 | 15580655 | 2.390 |
| *C09G1.2* | X | 15977762 | 15982600 | 1.020 |
| *B0395.3* | X | 16035055 | 16039514 | 1.013 |
| *ZK1073.2* | X | 16103643 | 16123903 | 1.178 |
| *W09B12.2* | X | 16363684 | 16363954 | 2.046 |
| *let-2* | X | 16380597 | 16389371 | 1.746 |
| *F01G12.13* | X | 16392315 | 16392511 | 2.038 |
| *T25D1.2* | X | 16515565 | 16519175 | 1.193 |
| *T27B1.4* | X | 16530821 | 16530955 | 1.531 |
| *pat-9* | X | 16536837 | 16540207 | 1.410 |
| *K08B5.7* | X | 16556944 | 16557168 | 3.053 |
| *ser-1* | X | 16579745 | 16587624 | 1.589 |
| *F59C12.3* | X | 16603364 | 16609524 | 1.033 |
| *F43B10.1* | X | 16638226 | 16662823 | 1.321 |
| *F43B10.5* | X | 16655352 | 16655598 | 1.618 |
| *F43B10.4* | X | 16655441 | 16655664 | 1.618 |
| *C10E2.5* | X | 16751864 | 16753673 | 1.038 |
| *mct-6* | X | 16767422 | 16770498 | 1.486 |
| *C10E2.7* | X | 16770158 | 16770302 | 1.920 |
| *cas-1* | X | 16807461 | 16813673 | 0.900 |
| *F41G4.5* | X | 16830673 | 16832740 | 1.933 |
| *F41G4.7* | X | 16833588 | 16836404 | 1.242 |
| *F41G4.8* | X | 16843355 | 16845024 | 1.369 |
| *F35B3.7* | X | 17035206 | 17042985 | 1.603 |
| *C30G4.4* | X | 17056925 | 17072188 | 0.839 |
| *acdh-7* | X | 17215018 | 17217105 | 1.079 |
| *mlc-2* | X | 17455779 | 17456586 | 2.442 |
| *C36E6.11* | X | 17457085 | 17457233 | 2.128 |
| *mlc-1* | X | 17459115 | 17460778 | 1.370 |
| *nhr-1* | X | 17518445 | 17528197 | 1.286 |
| *sup-10* | X | 17528344 | 17531980 | 1.512 |
| *F31A3.3* | X | 17532976 | 17534064 | 1.274 |
| *nlp-6* | X | 17662458 | 17665357 | 0.887 |
| *T23E7.8* | X | 17670486 | 17670770 | 2.168 |
| *T23E7.9* | X | 17670595 | 17670737 | 2.172 |
| *T23E7.2* | X | 17671029 | 17680793 | 1.469 |
