# Supplementary material for: DamID identifies targets of CEH-60/PBX that are associated with neuron development and muscle structure in Caenorhabditis elegans
Source: PLoS One. 2020 Dec 11;15(12):e0242939. doi: 10.1371/journal.pone.0242939 (PMC7732058; doi:10.1371/journal.pone.0242939)
Supplement: S2 Table — Start and stop represent the genomic positions on the specified chromosome of the specified open reading frame. Candidate gene targets are sorted by genomic position (i.e. chromosome number and start position). Log2FC is calculated as the log2 value of the ratio of average number of dam::ceh-60 reads over average number of gfp::dam reads. (DOCX) [file pone.0242939.s003.docx]

**S2 Table: 587 candidate gene targets of CEH-60 identified in L2 animals through DamID.** Start and stop represent the genomic positions on the specified chromosome of the specified open reading frame. Candidate gene targets are sorted by genomic position (*i.e.* chromosome number and start position). Log_2_FC is calculated as the log_2_ value of the ratio of average number of *dam::ceh-60* reads over average number of *gfp::dam* reads.

| **Gene** | **chr** | **Start** | **Stop** | **Log_2_FC** |
| --- | --- | --- | --- | --- |
| *nlp-40* | I | 11495 | 16837 | 1.467 |
| *Y65B4BL.6* | I | 520109 | 521554 | 1.327 |
| *ZC123.1* | I | 837996 | 841746 | 1.556 |
| *mig-1* | I | 948569 | 957404 | 1.033 |
| *npr-23* | I | 1062293 | 1065269 | 1.172 |
| *Y48G8AL.10* | I | 1136371 | 1154386 | 1.292 |
| *K12C11.5* | I | 1332698 | 1334074 | 2.343 |
| *tln-1* | I | 1721592 | 1740670 | 0.809 |
| *Y71G12B.5* | I | 1799717 | 1806986 | 1.080 |
| *C53H9.2* | I | 1832897 | 1834764 | 1.092 |
| *rla-1* | I | 2069086 | 2069587 | 0.935 |
| *cars-2* | I | 2629685 | 2632320 | 0.841 |
| *F32B5.6* | I | 2668160 | 2672434 | 0.948 |
| *Y71F9B.21* | I | 2703263 | 2703458 | 1.273 |
| *W03D8.2* | I | 2798175 | 2800878 | 0.905 |
| *lpd-6* | I | 3144410 | 3147793 | 1.178 |
| *cdap-2* | I | 3319540 | 3330673 | 1.127 |
| *C50F2.2* | I | 3881816 | 3888313 | 1.389 |
| *abf-1* | I | 3886111 | 3887080 | 1.091 |
| *C50F2.5* | I | 3895764 | 3897478 | 1.867 |
| *fkb-5* | I | 3898046 | 3899840 | 1.799 |
| *lrp-2* | I | 3935272 | 3953991 | 1.687 |
| *F28H1.4* | I | 3974063 | 3977351 | 1.411 |
| *aars-2* | I | 3983171 | 3986915 | 0.942 |
| *cpn-3* | I | 3987842 | 3988699 | 1.657 |
| *R12E2.13* | I | 4177305 | 4178447 | 1.370 |
| *cas-2* | I | 4190142 | 4193129 | 1.594 |
| *W09C3.2* | I | 4711414 | 4713572 | 2.388 |
| *dip-2* | I | 4934259 | 4949332 | 1.254 |
| *pat-10* | I | 5018770 | 5020166 | 0.810 |
| *C46H11.7* | I | 5042102 | 5043453 | 0.871 |
| *B0261.8* | I | 5271265 | 5272037 | 1.968 |
| *cpb-3* | I | 5788634 | 5791752 | 1.784 |
| *C34G6.9* | I | 5879139 | 5879491 | 1.563 |
| *C34G6.11* | I | 5879340 | 5879554 | 1.563 |
| *B0207.1* | I | 5965062 | 5967211 | 1.300 |
| *csn-2* | I | 6022660 | 6026874 | 1.375 |
| *ZK484.3* | I | 6068526 | 6070122 | 1.632 |
| *H27M09.7* | I | 6847134 | 6847308 | 0.931 |
| *H27M09.6* | I | 6847137 | 6847303 | 0.931 |
| *ubql-1* | I | 7021946 | 7029076 | 1.004 |
| *unc-15* | I | 7376604 | 7383197 | 1.140 |
| *chs-1* | I | 7554855 | 7559916 | 1.036 |
| *F30F8.1* | I | 7832692 | 7835453 | 1.515 |
| *R11A5.6* | I | 7867577 | 7867918 | 1.893 |
| *F10D11.5* | I | 8442695 | 8444934 | 1.824 |
| *D1081.16* | I | 8459041 | 8459254 | 2.099 |
| *D1081.15* | I | 8459050 | 8459331 | 2.099 |
| *ddx-52* | I | 8598658 | 8600923 | 1.186 |
| *K07A12.5* | I | 8693451 | 8695159 | 0.880 |
| *tin-13* | I | 8759204 | 8759788 | 1.132 |
| *lmn-1* | I | 8762986 | 8765633 | 1.218 |
| *F30A10.2* | I | 9479101 | 9480774 | 2.984 |
| *T05F1.5* | I | 9633183 | 9635945 | 0.873 |
| *C03C11.1* | I | 10014547 | 10015495 | 2.350 |
| *F16C3.4* | I | 10158951 | 10160034 | 1.871 |
| *fkh-10* | I | 10163475 | 10165135 | 1.108 |
| *T20F10.8* | I | 10290911 | 10292078 | 1.629 |
| *lonp-1* | I | 10676922 | 10682374 | 0.834 |
| *Y53H1B.2* | I | 11292662 | 11294197 | 1.283 |
| *col-66* | I | 11559446 | 11560565 | 1.594 |
| *mag-1* | I | 11912005 | 11912677 | 0.924 |
| *F14B6.t1* | I | 12229309 | 12229380 | 1.288 |
| *oac-35* | I | 12307731 | 12310406 | 4.315 |
| *T15D6.12* | I | 12399323 | 12401206 | 2.111 |
| *K11D2.1* | I | 12497467 | 12505010 | 1.315 |
| *clec-110* | I | 13204497 | 13205403 | 1.202 |
| *ZK1053.3* | I | 13246853 | 13249789 | 1.972 |
| *W05H12.2* | I | 13411594 | 13412879 | 0.857 |
| *W05H12.4* | I | 13411814 | 13412423 | 0.932 |
| *linc-1* | I | 13652524 | 13652727 | 1.031 |
| *C37A5.11* | I | 14171864 | 14172786 | 2.752 |
| *ZK849.1* | I | 14182787 | 14185717 | 1.504 |
| *clec-115* | I | 14246228 | 14247314 | 1.761 |
| *Y105E8A.2* | I | 14347514 | 14358326 | 1.621 |
| *unc-95* | I | 14381344 | 14384737 | 1.422 |
| *rpom-1* | I | 14552030 | 14565853 | 1.176 |
| *blos-9* | I | 14797660 | 14798705 | 2.052 |
| *metl-17* | I | 14839856 | 14843128 | 1.616 |
| *unc-54* | I | 14855901 | 14863573 | 1.601 |
| *tep-1* | I | 14993821 | 15001682 | 1.046 |
| *F33H2.8* | I | 15031145 | 15033039 | 1.284 |
| *rrn-2.1* | I | 15064301 | 15064453 | 1.060 |
| *col-69* | II | 303924 | 305530 | 0.904 |
| *Y39F10A.1* | II | 775333 | 776780 | 1.713 |
| *F46F5.3* | II | 801728 | 802693 | 2.984 |
| *K02E7.1* | II | 1083159 | 1083929 | 2.047 |
| *snf-3* | II | 1092006 | 1097899 | 0.823 |
| *oac-30* | II | 1262987 | 1266890 | 1.392 |
| *C08E3.13* | II | 1631995 | 1633850 | 1.027 |
| *str-22* | II | 1802107 | 1803302 | 3.592 |
| *sup-9* | II | 2684669 | 2686977 | 1.035 |
| *clec-120* | II | 2756021 | 2757018 | 2.192 |
| *F08D12.7* | II | 2767250 | 2769121 | 0.974 |
| *fbxb-105* | II | 2769670 | 2770888 | 2.212 |
| *lido-10* | II | 3002513 | 3003827 | 0.920 |
| *fbxc-50* | II | 3246316 | 3248174 | 3.488 |
| *nep-13* | II | 3282653 | 3287158 | 2.698 |
| *F12E12.3* | II | 3737334 | 3738471 | 2.269 |
| *T24E12.6* | II | 3760300 | 3761699 | 0.913 |
| *srx-112* | II | 3763447 | 3764586 | 2.489 |
| *F54D10.9* | II | 3829584 | 3831914 | 3.992 |
| *pqn-48* | II | 4038868 | 4043070 | 1.076 |
| *rpn-11* | II | 4043059 | 4044330 | 0.877 |
| *C16A11.4* | II | 4227046 | 4229157 | 1.780 |
| *hlh-1* | II | 4518560 | 4522523 | 1.751 |
| *scc-1* | II | 4698721 | 4700994 | 1.142 |
| *ctsa-1.1* | II | 4737617 | 4739906 | 1.484 |
| *nspd-5* | II | 4860064 | 4860411 | 3.580 |
| *shc-2* | II | 4969186 | 4988934 | 0.886 |
| *F59A6.2* | II | 5014699 | 5015324 | 1.596 |
| *drn-1* | II | 5270406 | 5271845 | 2.445 |
| *rsp-4* | II | 5387411 | 5388830 | 1.359 |
| *F56D1.14* | II | 5465988 | 5466135 | 1.361 |
| *F56D1.15* | II | 5466112 | 5466251 | 1.366 |
| *lec-5* | II | 5839639 | 5841134 | 1.683 |
| *rgs-3* | II | 6100060 | 6110122 | 1.332 |
| *upb-1* | II | 6288413 | 6290097 | 1.870 |
| *epg-5* | II | 6603441 | 6609356 | 0.828 |
| *T19D12.1* | II | 6666898 | 6673752 | 0.855 |
| *acer-1* | II | 6888553 | 6890418 | 1.259 |
| *kars-1* | II | 7070183 | 7072415 | 0.961 |
| *T07F8.1* | II | 7149248 | 7151760 | 0.947 |
| *R07G3.7* | II | 7610986 | 7613730 | 0.925 |
| *R07G3.13* | II | 7613344 | 7613490 | 1.097 |
| *R07G3.16* | II | 7623528 | 7623698 | 1.355 |
| *R07G3.10* | II | 7623578 | 7623816 | 1.355 |
| *pde-4* | II | 7627250 | 7651295 | 0.822 |
| *T05A6.15* | II | 7809203 | 7809409 | 1.351 |
| *rev-1* | II | 7900647 | 7904636 | 0.898 |
| *rpl-10* | II | 8143863 | 8144671 | 0.899 |
| *M05D6.10* | II | 8471880 | 8473522 | 0.793 |
| *T13H5.1* | II | 8487571 | 8509055 | 1.188 |
| *bcs-1* | II | 8571085 | 8572904 | 0.939 |
| *pyr-1* | II | 8651109 | 8658808 | 0.954 |
| *cup-16* | II | 8707155 | 8708860 | 1.823 |
| *F07H5.4* | II | 8786457 | 8787119 | 1.049 |
| *T14D7.4* | II | 8844702 | 8844924 | 0.855 |
| *T07D4.2* | II | 8869549 | 8874020 | 1.912 |
| *F44G4.6* | II | 9000872 | 9001793 | 1.442 |
| *rpl-32* | II | 9056499 | 9057111 | 1.747 |
| *Y9C2UA.1* | II | 9139651 | 9145352 | 1.040 |
| *aqp-2* | II | 9258913 | 9261994 | 1.210 |
| *C01G6.2* | II | 9267336 | 9268303 | 0.821 |
| *dnj-20* | II | 9617426 | 9624176 | 0.783 |
| *T09F3.6* | II | 10389765 | 10389975 | 1.423 |
| *cdl-1* | II | 10785452 | 10787272 | 1.415 |
| *col-81* | II | 11012397 | 11013820 | 1.110 |
| *unc-53* | II | 11058096 | 11089456 | 1.819 |
| *C09H10.12* | II | 11081164 | 11081309 | 2.207 |
| *F40F8.1* | II | 11127634 | 11129256 | 1.029 |
| *ptr-13* | II | 11157094 | 11162481 | 1.188 |
| *C47G2.21* | II | 11269038 | 11269187 | 2.691 |
| *unc-130* | II | 11281986 | 11283953 | 1.169 |
| *dnj-13* | II | 11545563 | 11547366 | 0.835 |
| *pcs-1* | II | 11574940 | 11577258 | 1.075 |
| *ints-7* | II | 11582849 | 11588536 | 0.888 |
| *ldh-1* | II | 11724204 | 11726533 | 0.987 |
| *alh-8* | II | 11728949 | 11731226 | 1.209 |
| *F13D12.8* | II | 11731165 | 11732594 | 0.985 |
| *Y38F1A.13* | II | 13020773 | 13021074 | 1.133 |
| *zyx-1* | II | 13056814 | 13071530 | 0.980 |
| *F32A11.8* | II | 13167847 | 13167984 | 1.756 |
| *mecr-1* | II | 13185987 | 13187351 | 2.005 |
| *E01G4.6* | II | 13482405 | 13485780 | 0.810 |
| *R06B9.5* | II | 13736349 | 13736933 | 1.334 |
| *arrd-13* | II | 13758408 | 13761233 | 0.906 |
| *Y39G8B.8* | II | 13993009 | 13993527 | 1.530 |
| *gur-4* | II | 14104773 | 14108072 | 1.562 |
| *Y54G11A.11* | II | 14349783 | 14350795 | 0.889 |
| *mltn-9* | II | 14616068 | 14622612 | 0.922 |
| *smg-9* | II | 14746890 | 14752489 | 2.837 |
| *ver-2* | III | 148087 | 150197 | 2.262 |
| *F40G9.5* | III | 183231 | 184915 | 1.117 |
| *F42G9.10* | III | 766504 | 766653 | 1.154 |
| *F23H11.2* | III | 895786 | 900158 | 0.814 |
| *Y22D7AL.16* | III | 1662104 | 1665152 | 1.113 |
| *Y22D7AR.7* | III | 1683630 | 1688099 | 0.898 |
| *srd-69* | III | 2362948 | 2366278 | 0.945 |
| *ppfr-4* | III | 2619936 | 2622709 | 0.783 |
| *phf-15* | III | 3322962 | 3331727 | 1.125 |
| *C32A3.7* | III | 3621520 | 3621644 | 0.883 |
| *H38K22.8* | III | 4317339 | 4317381 | 2.050 |
| *C28A5.6* | III | 4423711 | 4429499 | 0.903 |
| *rps-1* | III | 4475918 | 4476887 | 0.825 |
| *ins-17* | III | 4476888 | 4477583 | 0.897 |
| *dnj-18* | III | 4697987 | 4699453 | 1.142 |
| *B0393.4* | III | 4759744 | 4763609 | 1.075 |
| *mel-28* | III | 4786080 | 4792391 | 1.126 |
| *acbp-7* | III | 4840187 | 4841024 | 1.182 |
| *mlc-3* | III | 5565025 | 5567382 | 1.012 |
| *ddx-15* | III | 5589036 | 5591718 | 1.239 |
| *mlp-1* | III | 5971530 | 5973664 | 1.032 |
| *T12A2.1* | III | 6251811 | 6271282 | 1.846 |
| *C56G2.9* | III | 6342481 | 6343287 | 0.924 |
| *dlc-1* | III | 6462907 | 6463801 | 1.557 |
| *pdfr-1* | III | 6632841 | 6652083 | 1.157 |
| *K07E12.3* | III | 6744662 | 6744790 | 1.768 |
| *dig-1* | III | 6746308 | 6794764 | 1.115 |
| *C06E8.5* | III | 7002301 | 7005450 | 1.236 |
| *R01H2.4* | III | 7061111 | 7064275 | 1.959 |
| *F56C9.10* | III | 7329714 | 7335316 | 0.908 |
| *F56C9.12* | III | 7337892 | 7338002 | 1.471 |
| *C07H6.4* | III | 7499677 | 7503241 | 1.578 |
| *ceh-13* | III | 7555617 | 7558208 | 1.033 |
| *egl-45* | III | 7824377 | 7828626 | 0.981 |
| *ZK688.9* | III | 7881113 | 7883003 | 1.419 |
| *lin-36* | III | 8016648 | 8020873 | 1.306 |
| *plk-1* | III | 8101348 | 8104076 | 0.830 |
| *hsp-110* | III | 8443917 | 8446920 | 1.000 |
| *rpn-3* | III | 8447353 | 8449346 | 1.502 |
| *pat-2* | III | 8818899 | 8825270 | 0.897 |
| *far-1* | III | 9080371 | 9081179 | 1.591 |
| *F02A9.9* | III | 9081466 | 9081595 | 1.815 |
| *far-2* | III | 9081679 | 9082511 | 1.852 |
| *C40H1.3* | III | 9327307 | 9329366 | 1.314 |
| *emb-9* | III | 9336881 | 9344447 | 1.477 |
| *C05B5.2* | III | 9999819 | 10000528 | 1.110 |
| *fbxa-155* | III | 10007966 | 10010177 | 1.985 |
| *vglu-3* | III | 10300293 | 10303423 | 3.305 |
| *atx-2* | III | 10461389 | 10468051 | 0.790 |
| *unc-49* | III | 10520676 | 10532705 | 1.685 |
| *sca-1* | III | 10811906 | 10816844 | 1.601 |
| *rsa-2* | III | 11063479 | 11068753 | 1.417 |
| *tbx-37* | III | 11224800 | 11227125 | 1.200 |
| *T28D6.4* | III | 11360456 | 11366214 | 1.190 |
| *mrt-2* | III | 11747042 | 11750160 | 1.950 |
| *npp-16* | III | 11915891 | 11917876 | 1.081 |
| *Y79H2A.2* | III | 12028969 | 12030769 | 0.862 |
| *Y111B2A.25* | III | 12528287 | 12536848 | 1.074 |
| *Y37D8A.2* | III | 12820141 | 12825651 | 1.623 |
| *Y39E4B.10* | III | 13143373 | 13152858 | 2.465 |
| *T05D4.2* | III | 13561729 | 13563082 | 1.112 |
| *aldo-1* | III | 13563346 | 13564990 | 1.226 |
| *T05C7.t1* | IV | 66982 | 67052 | 3.261 |
| *fln-1* | IV | 359204 | 376062 | 0.791 |
| *T07A9.14* | IV | 386699 | 387949 | 1.291 |
| *kvs-5* | IV | 736675 | 753316 | 1.543 |
| *C44B12.9* | IV | 1103541 | 1104609 | 1.302 |
| *ost-1* | IV | 1107396 | 1110856 | 1.308 |
| *F38A1.17* | IV | 1260207 | 1260519 | 1.312 |
| *K08D12.3* | IV | 1710265 | 1711127 | 0.791 |
| *Y41D4A.7* | IV | 1752270 | 1761647 | 1.286 |
| *Y38F2AR.10* | IV | 2396257 | 2399420 | 1.904 |
| *Y54G2A.73* | IV | 3000541 | 3008892 | 1.217 |
| *unc-33* | IV | 3516819 | 3526602 | 1.062 |
| *F37C4.8* | IV | 3878547 | 3881187 | 1.205 |
| *F15E6.9* | IV | 4293620 | 4299083 | 1.709 |
| *F19C7.8* | IV | 4610576 | 4638095 | 1.649 |
| *F29B9.1* | IV | 4665251 | 4667293 | 1.160 |
| *ogdh-1* | IV | 4696982 | 4704011 | 1.181 |
| *Y17G9B.5* | IV | 4750240 | 4751491 | 1.547 |
| *rme-2* | IV | 5469961 | 5473174 | 1.224 |
| *drp-1* | IV | 5537981 | 5541365 | 1.292 |
| *B0350.79* | IV | 5980619 | 5980754 | 0.786 |
| *21ur-14209* | IV | 5981091 | 5981111 | 1.055 |
| *K08B4.43* | IV | 6103136 | 6103274 | 3.233 |
| *T05A12.3* | IV | 6883576 | 6887662 | 1.425 |
| *ZC477.4* | IV | 7098542 | 7098784 | 0.782 |
| *ZC477.16* | IV | 7098571 | 7098933 | 0.782 |
| *rps-8* | IV | 7312981 | 7313864 | 1.170 |
| *ima-3* | IV | 7558726 | 7561664 | 1.199 |
| *C50F7.6* | IV | 7720566 | 7723199 | 1.114 |
| *C06A6.14* | IV | 7845656 | 7845868 | 1.180 |
| *C49H3.6* | IV | 7903740 | 7907083 | 1.101 |
| *K07H8.5* | IV | 8271054 | 8272524 | 1.200 |
| *K07H8.9* | IV | 8285175 | 8286566 | 1.157 |
| *F42A9.18* | IV | 8605512 | 8607840 | 1.692 |
| *21ur-9384* | IV | 8721667 | 8721687 | 1.558 |
| *C27B7.5* | IV | 8901646 | 8903177 | 1.735 |
| *C27B7.6* | IV | 8903222 | 8906205 | 1.312 |
| *F49C12.7* | IV | 9309108 | 9312927 | 1.766 |
| *rpn-7* | IV | 9316639 | 9318632 | 1.343 |
| *elo-1* | IV | 9526359 | 9528350 | 0.837 |
| *npp-1* | IV | 9858488 | 9862118 | 0.873 |
| *spe-29* | IV | 9933910 | 9934285 | 1.796 |
| *dhp-2* | IV | 9978122 | 9980770 | 1.800 |
| *frk-1* | IV | 10043183 | 10045739 | 1.125 |
| *dhs-12* | IV | 10145249 | 10146222 | 1.180 |
| *tsp-12* | IV | 10149166 | 10150602 | 0.907 |
| *ttr-54* | IV | 10155775 | 10156771 | 1.474 |
| *K04D7.17* | IV | 10176491 | 10176675 | 0.815 |
| *acs-18* | IV | 10310772 | 10313670 | 2.048 |
| *C02F4.10* | IV | 10503673 | 10503934 | 0.883 |
| *glb-19* | IV | 10642912 | 10654147 | 1.955 |
| *ugt-54* | IV | 10761597 | 10764496 | 0.826 |
| *F01G4.4* | IV | 11143345 | 11145923 | 0.999 |
| *F01G4.6* | IV | 11146940 | 11149504 | 1.124 |
| *pfd-1* | IV | 11150113 | 11150930 | 2.101 |
| *C10C6.11* | IV | 11474031 | 11474252 | 1.023 |
| *C10C6.8* | IV | 11474059 | 11474191 | 1.023 |
| *rib-1* | IV | 11578757 | 11584066 | 0.936 |
| *ZK809.14* | IV | 11652109 | 11652251 | 0.924 |
| *T12A7.12* | IV | 11748939 | 11749074 | 1.400 |
| *dyf-18* | IV | 11798667 | 11801088 | 1.758 |
| *nhx-9* | IV | 11930872 | 11935718 | 1.256 |
| *ZK829.9* | IV | 11970534 | 11972789 | 0.910 |
| *ZK617.25* | IV | 12006208 | 12006357 | 1.974 |
| *ZK617.23* | IV | 12010399 | 12010681 | 0.905 |
| *ZK617.8* | IV | 12012388 | 12012667 | 1.513 |
| *ZK617.18* | IV | 12012463 | 12012876 | 1.513 |
| *spe-17* | IV | 12013420 | 12014045 | 1.124 |
| *rbm-34* | IV | 12085423 | 12087060 | 0.789 |
| *col-129* | IV | 12108672 | 12110275 | 1.783 |
| *gcy-18* | IV | 12860137 | 12865935 | 1.030 |
| *lev-1* | IV | 13174157 | 13179457 | 1.476 |
| *memi-2* | IV | 13399068 | 13400506 | 1.125 |
| *21ur-14362* | IV | 13869998 | 13870018 | 1.796 |
| *21ur-5470* | IV | 14224943 | 14224963 | 1.257 |
| *Y57G11B.5* | IV | 14574819 | 14576580 | 1.942 |
| *Y57G11C.33* | IV | 14657081 | 14658165 | 1.563 |
| *mrps-7* | IV | 14766773 | 14767730 | 0.881 |
| *Y105C5A.14* | IV | 15646970 | 15648298 | 1.581 |
| *Y105C5B.9* | IV | 15930581 | 15931813 | 1.387 |
| *Y105C5B.1420* | IV | 16156908 | 16165267 | 0.895 |
| *C35D6.3* | IV | 16347168 | 16347430 | 2.058 |
| *Y116A8C.9* | IV | 16939804 | 16946910 | 2.081 |
| *T02D1.4* | IV | 17399776 | 17402336 | 1.110 |
| *4R79.2* | IV | 17480396 | 17483332 | 1.050 |
| *npr-5* | V | 276989 | 281716 | 1.177 |
| *nhr-58* | V | 532290 | 534064 | 0.987 |
| *C14C6.12* | V | 570571 | 571205 | 1.627 |
| *icl-1* | V | 724207 | 728103 | 1.246 |
| *madd-2* | V | 1223901 | 1235445 | 1.077 |
| *nphp-2* | V | 1895797 | 1906560 | 1.277 |
| *srp-3* | V | 1908855 | 1910692 | 1.583 |
| *comt-4* | V | 2049223 | 2050172 | 0.836 |
| *C29G2.7* | V | 2575662 | 2576717 | 1.073 |
| *ketn-1* | V | 2781943 | 2807934 | 1.063 |
| *srh-36* | V | 2819581 | 2821668 | 2.494 |
| *C31B8.12* | V | 2925193 | 2927722 | 2.442 |
| *str-66* | V | 3103128 | 3104234 | 2.264 |
| *str-242* | V | 3122969 | 3124222 | 3.055 |
| *srab-11* | V | 3145203 | 3147032 | 3.274 |
| *srt-63* | V | 3254602 | 3256151 | 1.858 |
| *T28A11.20* | V | 3271395 | 3273256 | 1.295 |
| *T28A11.3* | V | 3275333 | 3276110 | 1.539 |
| *F35F10.6* | V | 3287995 | 3289385 | 2.066 |
| *srx-122* | V | 3296108 | 3297864 | 1.157 |
| *C17B7.4* | V | 3339348 | 3340092 | 2.040 |
| *C17B7.3* | V | 3342804 | 3343865 | 1.649 |
| *arrd-21* | V | 3389330 | 3390952 | 0.882 |
| *T20D4.15* | V | 3393544 | 3394744 | 2.301 |
| *T20D4.10* | V | 3400252 | 3401063 | 1.799 |
| *T20D4.20* | V | 3401645 | 3402356 | 1.853 |
| *T20D4.9* | V | 3402631 | 3405161 | 1.764 |
| *arrd-22* | V | 3410520 | 3412858 | 0.842 |
| *str-113* | V | 3664871 | 3666987 | 1.090 |
| *srx-63* | V | 3931626 | 3933394 | 3.010 |
| *nlp-34* | V | 3978836 | 3979134 | 3.998 |
| *Y45G5AM.6* | V | 4147550 | 4152296 | 0.811 |
| *Y45G5AM.3* | V | 4164554 | 4166825 | 1.127 |
| *srt-35* | V | 4261485 | 4264616 | 1.200 |
| *D2063.1* | V | 4327121 | 4328736 | 1.184 |
| *skr-7* | V | 4422406 | 4423065 | 1.365 |
| *unc-62* | V | 4497463 | 4511449 | 0.998 |
| *Y61A9LA.12* | V | 4553872 | 4554498 | 0.834 |
| *F54D11.3* | V | 4635400 | 4637145 | 1.561 |
| *C18G1.1* | V | 4777960 | 4780019 | 1.684 |
| *B0238.11* | V | 5269485 | 5270744 | 0.947 |
| *dmsr-7* | V | 5405358 | 5408386 | 1.324 |
| *K09H11.9* | V | 5714760 | 5716581 | 1.006 |
| *F44E7.15* | V | 5780500 | 5780645 | 2.258 |
| *mir-253* | V | 5780510 | 5780616 | 2.258 |
| *W06H8.10* | V | 6204165 | 6204452 | 1.914 |
| *W06H8.2* | V | 6206755 | 6208551 | 1.252 |
| *scd-2* | V | 6633269 | 6639665 | 1.243 |
| *T25F10.9* | V | 6757042 | 6757176 | 0.796 |
| *bbs-8* | V | 6762476 | 6765072 | 1.699 |
| *clik-1* | V | 6765560 | 6768732 | 1.028 |
| *srh-30* | V | 6769662 | 6771704 | 1.746 |
| *F09G2.10* | V | 7175054 | 7175254 | 2.628 |
| *C03G6.5* | V | 7365251 | 7366204 | 2.461 |
| *ddo-2* | V | 7433401 | 7435650 | 0.813 |
| *C50E3.12* | V | 7620504 | 7622061 | 2.371 |
| *ZK742.4* | V | 7810228 | 7811785 | 1.301 |
| *memo-1* | V | 7844343 | 7847164 | 1.689 |
| *B0507.2* | V | 8774332 | 8776630 | 1.106 |
| *F21C10.7* | V | 9112767 | 9122180 | 1.167 |
| *spl-2* | V | 9144630 | 9146899 | 1.233 |
| *str-177* | V | 9197751 | 9198987 | 1.063 |
| *asp-2* | V | 9203077 | 9204734 | 1.528 |
| *glo-4* | V | 9234610 | 9243221 | 1.039 |
| *F44A2.5* | V | 9297632 | 9302158 | 1.039 |
| *gsnl-1* | V | 9497042 | 9499372 | 2.113 |
| *mdf-1* | V | 9541782 | 9544226 | 0.960 |
| *ztf-9* | V | 9691039 | 9692159 | 1.785 |
| *anmt-3* | V | 9956879 | 9958331 | 1.701 |
| *C27H6.8* | V | 9986330 | 9987788 | 1.056 |
| *uda-1* | V | 10004755 | 10007482 | 0.776 |
| *C51E3.9* | V | 10145905 | 10148977 | 0.843 |
| *ZK856.7* | V | 10201856 | 10202610 | 1.942 |
| *Y32F6A.5* | V | 10446534 | 10453781 | 1.960 |
| *F22E12.1* | V | 10453748 | 10457347 | 0.919 |
| *C29A12.9* | V | 10819943 | 10820145 | 3.461 |
| *F32D8.1* | V | 10880837 | 10883399 | 1.016 |
| *F17C11.20* | V | 10968312 | 10969544 | 1.553 |
| *C06H2.7* | V | 11143062 | 11144758 | 1.340 |
| *C03E10.8* | V | 11281583 | 11281725 | 1.012 |
| *C03E10.9* | V | 11281951 | 11282097 | 1.206 |
| *T03F7.11* | V | 11307918 | 11308060 | 1.825 |
| *des-2* | V | 11415342 | 11420293 | 1.550 |
| *C55A6.10* | V | 11505352 | 11508271 | 1.489 |
| *ttll-5* | V | 11509068 | 11512318 | 1.146 |
| *C55A6.3* | V | 11512313 | 11513436 | 0.795 |
| *F46F3.6* | V | 11675086 | 11675304 | 1.361 |
| *F46F3.15* | V | 11675149 | 11675358 | 1.361 |
| *ceh-32* | V | 11701764 | 11705400 | 1.260 |
| *W05E10.16* | V | 11705275 | 11705421 | 1.260 |
| *vab-8* | V | 12213327 | 12225935 | 1.115 |
| *myo-3* | V | 12226816 | 12234343 | 1.599 |
| *hrg-2* | V | 12409369 | 12410513 | 1.465 |
| *R186.3* | V | 12966000 | 12967210 | 2.382 |
| *C34D1.6* | V | 13226065 | 13226288 | 1.473 |
| *cpz-2* | V | 13381189 | 13383077 | 1.153 |
| *swan-1* | V | 13804395 | 13807237 | 0.800 |
| *stdh-1* | V | 13906492 | 13908135 | 1.195 |
| *D1086.3* | V | 14092792 | 14093986 | 2.294 |
| *D1086.12* | V | 14101186 | 14102821 | 1.851 |
| *H39E23.3* | V | 14107811 | 14110151 | 1.606 |
| *acs-10* | V | 14146499 | 14148784 | 1.119 |
| *F56A12.11* | V | 14359109 | 14359253 | 1.423 |
| *gar-3* | V | 14564752 | 14579719 | 1.076 |
| *R11H6.4* | V | 14609943 | 14610705 | 0.974 |
| *F43D2.11* | V | 14618744 | 14619091 | 1.686 |
| *F43D2.13* | V | 14618842 | 14619106 | 1.686 |
| *R08A2.2* | V | 14714207 | 14715720 | 1.612 |
| *Y50E8A.6* | V | 14738542 | 14741326 | 0.806 |
| *fib-1* | V | 15000653 | 15002273 | 1.850 |
| *nmr-2* | V | 15006412 | 15011660 | 0.936 |
| *cng-1* | V | 15025740 | 15031473 | 3.422 |
| *T09F5.20* | V | 15146387 | 15146861 | 0.873 |
| *cyn-7* | V | 15186582 | 15187787 | 1.101 |
| *linc-108* | V | 15565170 | 15566425 | 1.179 |
| *F53E4.1* | V | 15652438 | 15668300 | 0.987 |
| *nhr-283* | V | 15774946 | 15776676 | 2.435 |
| *F35E8.9* | V | 15916334 | 15917558 | 1.296 |
| *fbxa-100* | V | 16060526 | 16062093 | 2.513 |
| *srh-98* | V | 16235171 | 16235747 | 2.701 |
| *srv-35* | V | 16378892 | 16380488 | 1.420 |
| *F26D2.3* | V | 16407963 | 16410270 | 1.335 |
| *srd-21* | V | 16629900 | 16631155 | 1.873 |
| *phy-3* | V | 16833316 | 16839141 | 1.095 |
| *tni-3* | V | 16833638 | 16835144 | 2.647 |
| *Y102A5C.38* | V | 16994939 | 16998771 | 2.685 |
| *F59A1.13* | V | 17687211 | 17690994 | 2.907 |
| *cutl-4* | V | 17733568 | 17739899 | 1.463 |
| *twk-36* | V | 17743651 | 17747059 | 1.520 |
| *srh-178* | V | 18478467 | 18480331 | 1.891 |
| *ttr-24* | V | 18555387 | 18556643 | 1.003 |
| *srh-208* | V | 19317362 | 19318500 | 3.111 |
| *C25F9.2* | V | 19421471 | 19426774 | 1.484 |
| *Y43F8C.7* | V | 19641292 | 19644083 | 0.885 |
| *Y43F8C.22* | V | 19643050 | 19643187 | 1.262 |
| *fbxa-131* | V | 19738417 | 19739393 | 1.455 |
| *M162.5* | V | 19761051 | 19766763 | 0.921 |
| *lgc-55* | V | 20023046 | 20036667 | 1.792 |
| *B0250.8* | V | 20469703 | 20470485 | 5.918 |
| *F46B3.2* | V | 20598508 | 20599492 | 1.765 |
| *F46B3.20* | V | 20618910 | 20621966 | 1.369 |
| *F38A6.4* | V | 20773743 | 20775369 | 1.174 |
| *clec-264* | V | 20847702 | 20850666 | 1.494 |
| *Y73B3A.1* | X | 42728 | 46924 | 1.398 |
| *T08D2.5* | X | 178242 | 179376 | 3.636 |
| *B0310.1* | X | 493211 | 497510 | 1.215 |
| *B0310.3* | X | 510818 | 520880 | 1.264 |
| *cdo-1* | X | 862268 | 863851 | 2.117 |
| *C05D9.3* | X | 1111903 | 1116794 | 0.855 |
| *tmc-1* | X | 1163153 | 1177172 | 0.830 |
| *F09E10.1* | X | 1502860 | 1503278 | 1.360 |
| *F48B9.11* | X | 2155659 | 2155798 | 2.336 |
| *K02E10.7* | X | 2501003 | 2508549 | 1.424 |
| *R11B5.10* | X | 2518676 | 2518739 | 1.757 |
| *F52H2.7* | X | 2566557 | 2572653 | 1.217 |
| *aqp-7* | X | 3025505 | 3026976 | 1.621 |
| *pccb-1* | X | 3089496 | 3092457 | 1.188 |
| *tba-9* | X | 3252963 | 3256265 | 1.073 |
| *sul-1* | X | 3267294 | 3270906 | 1.117 |
| *acp-1* | X | 3382868 | 3385547 | 1.492 |
| *ZK813.5* | X | 3448744 | 3451222 | 1.458 |
| *T27A10.6* | X | 3588858 | 3596497 | 1.010 |
| *C02F12.9* | X | 3696903 | 3700807 | 1.256 |
| *tag-18* | X | 3731894 | 3733457 | 1.047 |
| *F47F2.8* | X | 3899244 | 3899407 | 1.202 |
| *adt-3* | X | 3931631 | 3937897 | 1.293 |
| *lin-18* | X | 3958497 | 3961907 | 1.212 |
| *C16B8.6* | X | 3971863 | 3972002 | 2.466 |
| *F09F9.13* | X | 4110022 | 4110153 | 1.681 |
| *F09F9.10* | X | 4110058 | 4110231 | 1.681 |
| *C24A8.5* | X | 4328400 | 4328916 | 1.529 |
| *F14H12.8* | X | 4363607 | 4365069 | 1.408 |
| *lst-2* | X | 4378004 | 4383366 | 0.897 |
| *mltn-13* | X | 4442152 | 4448383 | 1.310 |
| *F43C9.1* | X | 4793266 | 4796158 | 1.444 |
| *act-4* | X | 4960553 | 4964416 | 1.103 |
| *ZC449.4* | X | 5028184 | 5029568 | 1.757 |
| *T03G11.19* | X | 5193554 | 5193755 | 1.024 |
| *C25F6.10* | X | 5475197 | 5475393 | 1.241 |
| *C54G7.10* | X | 5541165 | 5541307 | 0.931 |
| *F13D11.17* | X | 5819105 | 5819343 | 1.102 |
| *F49E10.15* | X | 5859334 | 5859532 | 1.394 |
| *F49E10.17* | X | 5859429 | 5859575 | 1.394 |
| *C45B2.1* | X | 6086124 | 6086567 | 2.713 |
| *lrch-1* | X | 6208761 | 6213911 | 1.173 |
| *C14F11.22* | X | 6211755 | 6211903 | 1.719 |
| *C14F11.17* | X | 6224890 | 6225159 | 1.303 |
| *C14F11.32* | X | 6224890 | 6225090 | 1.303 |
| *got-2.2* | X | 6240955 | 6242709 | 0.838 |
| *lev-9* | X | 6281847 | 6291732 | 1.313 |
| *T07H6.7* | X | 6291493 | 6291695 | 2.031 |
| *T22E5.12* | X | 6402683 | 6402881 | 0.925 |
| *mup-2* | X | 6408470 | 6410359 | 1.788 |
| *T22E5.10* | X | 6410539 | 6410672 | 2.020 |
| *asp-14* | X | 6444189 | 6446664 | 1.212 |
| *R03E9.10* | X | 6743978 | 6744311 | 1.674 |
| *R03E9.7* | X | 6744272 | 6744601 | 1.488 |
| *gei-15* | X | 6820005 | 6823268 | 1.275 |
| *F41B4.2* | X | 6831251 | 6835266 | 1.031 |
| *unc-6* | X | 6889689 | 6897147 | 0.844 |
| *R173.5* | X | 7018796 | 7018850 | 1.537 |
| *lam-2* | X | 7143918 | 7151235 | 1.045 |
| *C54D1.9* | X | 7151458 | 7151745 | 1.288 |
| *syd-9* | X | 7218109 | 7226825 | 1.030 |
| *F46H5.7* | X | 7238666 | 7242983 | 1.362 |
| *mec-7* | X | 7774859 | 7776689 | 1.220 |
| *pcyt-2.2* | X | 7884288 | 7887479 | 0.923 |
| *C39D10.7* | X | 7907955 | 7913930 | 1.209 |
| *F55E10.1* | X | 8341975 | 8342286 | 1.018 |
| *tnt-2* | X | 8721557 | 8723552 | 1.014 |
| *asns-2* | X | 8748434 | 8750969 | 1.505 |
| *C28G1.t4* | X | 8838338 | 8838409 | 2.531 |
| *ttr-38* | X | 9312648 | 9314488 | 1.980 |
| *dyf-7* | X | 9698678 | 9701679 | 1.006 |
| *rrc-1* | X | 9823752 | 9831249 | 1.106 |
| *ipla-2* | X | 9833352 | 9838039 | 0.889 |
| *R07B1.23* | X | 9842186 | 9842329 | 1.601 |
| *vab-15* | X | 9847275 | 9849518 | 1.049 |
| *F19C6.3* | X | 10005733 | 10008755 | 1.925 |
| *R07E3.4* | X | 10330547 | 10331900 | 0.972 |
| *acr-8* | X | 10412725 | 10416630 | 1.177 |
| *sdn-1* | X | 10589780 | 10593334 | 0.773 |
| *dgn-1* | X | 10920533 | 10928998 | 1.185 |
| *T21B6.3* | X | 10945160 | 10948970 | 1.804 |
| *F08G12.3* | X | 11300283 | 11302094 | 0.800 |
| *F42E11.6* | X | 11369047 | 11369158 | 1.626 |
| *F42E11.3* | X | 11374599 | 11375609 | 2.517 |
| *lin-14* | X | 11465557 | 11484368 | 0.822 |
| *C35C5.15* | X | 11560203 | 11560460 | 1.284 |
| *C35C5.17* | X | 11560220 | 11560467 | 1.284 |
| *gei-3* | X | 12795216 | 12813947 | 0.807 |
| *K02A4.14* | X | 12876770 | 12877035 | 1.065 |
| *K02A4.8* | X | 12878090 | 12878222 | 1.337 |
| *C34E7.4* | X | 12932205 | 12934978 | 0.951 |
| *C49F8.10* | X | 13359013 | 13359073 | 1.650 |
| *F02D10.6* | X | 13449586 | 13451678 | 1.824 |
| *F54B11.8* | X | 13602200 | 13603918 | 2.812 |
| *C31E10.4* | X | 13989496 | 13990180 | 1.818 |
| *C04C11.19* | X | 14039816 | 14040019 | 1.685 |
| *C33G3.10* | X | 14054048 | 14054383 | 1.996 |
| *C33G3.11* | X | 14054785 | 14054909 | 1.869 |
| *madd-3* | X | 14284775 | 14296658 | 1.034 |
| *syg-2* | X | 14654972 | 14662841 | 1.350 |
| *F40E10.14* | X | 14684549 | 14684644 | 1.060 |
| *Y70D2A.3* | X | 14920660 | 14920783 | 1.698 |
| *ucr-2.2* | X | 15173904 | 15178145 | 1.236 |
| *C02C6.8* | X | 15575526 | 15575801 | 1.186 |
| *C02C6.6* | X | 15575555 | 15575757 | 1.186 |
| *ent-2* | X | 15601067 | 15603763 | 0.813 |
| *acox-1.6* | X | 15834052 | 15838546 | 1.020 |
| *F31B9.5* | X | 15912383 | 15912529 | 1.981 |
| *F09C8.2* | X | 16140507 | 16147022 | 0.865 |
| *R11.7* | X | 16208814 | 16208914 | 2.302 |
| *let-2* | X | 16380597 | 16389371 | 1.552 |
| *F01G12.13* | X | 16392315 | 16392511 | 1.422 |
| *pat-9* | X | 16536837 | 16540207 | 1.216 |
| *ser-1* | X | 16579745 | 16587624 | 0.971 |
| *F43B10.1* | X | 16638226 | 16662823 | 1.481 |
| *mct-6* | X | 16767422 | 16770498 | 1.366 |
| *linc-100* | X | 16775220 | 16775505 | 1.128 |
| *cas-1* | X | 16807461 | 16813673 | 0.967 |
| *sto-5* | X | 16816260 | 16839975 | 1.804 |
| *F35B3.4* | X | 17019616 | 17020958 | 0.945 |
| *acdh-7* | X | 17215018 | 17217105 | 1.104 |
| *sup-10* | X | 17528344 | 17531980 | 1.220 |
| *F31A3.3* | X | 17532976 | 17534064 | 1.513 |
| *T23E7.8* | X | 17670486 | 17670770 | 1.519 |
| *T23E7.9* | X | 17670595 | 17670737 | 1.534 |
