# Supplementary material for: DamID identifies targets of CEH-60/PBX that are associated with neuron development and muscle structure in Caenorhabditis elegans
Source: PLoS One. 2020 Dec 11;15(12):e0242939. doi: 10.1371/journal.pone.0242939 (PMC7732058; doi:10.1371/journal.pone.0242939)
Supplement: S3 Table — Complete list of biological processes overrepresented in CEH-60 DamID targets. Observed and expected columns represent the number of genes associated with the specified ontology term and the expected number in a random collection of genes for the C. elegans genome. Fold enrichment represents the number of times the specified process is overrepresented in the dataset. FDR p value represents the false discovery rate corrected p value for the specified biological process. GO terms were sorted by FDR p value. Gene ontology analysis was carried out with PANTHER 15 using a statistical overrepresentation test for biological processes (complete). (DOCX) [file pone.0242939.s004.docx]

**S3 Table:** **Complete gene ontology analysis of 872 DamID gene targets in young adult animals.** Complete list of biological processes overrepresented in CEH-60 DamID targets. Observed and expected columns represent the number of genes associated with the specified ontology term and the expected number in a random collection of genes for the *C. elegans* genome. Fold enrichment represents the number of times the specified process is overrepresented in the dataset. FDR p value represents the false discovery rate corrected p-value for the specified biological process. GO terms were sorted by FDR p value. Gene ontology analysis was carried out with PANTHER 15 using statistical overrepresentation test for biological processes (complete).

| **Biological process** | **Observed** | **Expected** | **Fold enrichment** | **raw P value** | **FDR p value** |
| --- | --- | --- | --- | --- | --- |
| cellular process | 664 | 471.34 | 1.41 | 1.95E-24 | 4.09E-21 |
| cellular component organization | 225 | 122.19 | 1.84 | 2.80E-17 | 4.40E-14 |
| cellular component organization or biogenesis | 235 | 131.92 | 1.78 | 1.44E-16 | 1.82E-13 |
| metabolic process | 448 | 320.53 | 1.4 | 7.18E-14 | 7.53E-11 |
| cellular metabolic process | 385 | 268.53 | 1.43 | 4.26E-13 | 3.83E-10 |
| organic substance metabolic process | 405 | 287.14 | 1.41 | 7.39E-13 | 5.82E-10 |
| nitrogen compound metabolic process | 356 | 244.87 | 1.45 | 8.97E-13 | 6.27E-10 |
| macromolecule metabolic process | 320 | 216.53 | 1.48 | 3.19E-12 | 1.82E-09 |
| primary metabolic process | 383 | 271.01 | 1.41 | 3.02E-12 | 1.90E-09 |
| cellular macromolecule metabolic process | 249 | 164.88 | 1.51 | 3.30E-10 | 1.73E-07 |
| multicellular organismal process | 240 | 164.38 | 1.46 | 1.20E-08 | 5.81E-06 |
| organelle organization | 141 | 85.18 | 1.66 | 3.17E-08 | 1.33E-05 |
| biological regulation | 399 | 307.1 | 1.3 | 2.99E-08 | 1.34E-05 |
| cellular component assembly | 88 | 45.82 | 1.92 | 5.80E-08 | 2.28E-05 |
| cellular component biogenesis | 102 | 56.33 | 1.81 | 6.74E-08 | 2.50E-05 |
| cytoskeleton organization | 57 | 24.58 | 2.32 | 7.49E-08 | 2.62E-05 |
| locomotion | 60 | 26.71 | 2.25 | 8.24E-08 | 2.73E-05 |
| organonitrogen compound metabolic process | 261 | 187.19 | 1.39 | 1.06E-07 | 3.34E-05 |
| localization | 217 | 150.1 | 1.45 | 1.40E-07 | 4.21E-05 |
| cellular component morphogenesis | 38 | 13.57 | 2.8 | 1.48E-07 | 4.25E-05 |
| cellular nitrogen compound metabolic process | 170 | 111.53 | 1.52 | 2.01E-07 | 5.51E-05 |
| muscle structure development | 23 | 5.9 | 3.9 | 3.59E-07 | 9.43E-05 |
| regulation of biological process | 364 | 282.59 | 1.29 | 4.12E-07 | 1.04E-04 |
| cellular protein metabolic process | 185 | 125.88 | 1.47 | 5.19E-07 | 1.26E-04 |
| regulation of biological quality | 102 | 59.25 | 1.72 | 7.71E-07 | 1.74E-04 |
| protein metabolic process | 214 | 150.96 | 1.42 | 7.54E-07 | 1.76E-04 |
| organic cyclic compound metabolic process | 139 | 90.86 | 1.53 | 2.48E-06 | 5.38E-04 |
| actin filament-based process | 30 | 10.66 | 2.82 | 2.68E-06 | 5.63E-04 |
| movement of cell or subcellular component | 47 | 21.1 | 2.23 | 2.80E-06 | 5.68E-04 |
| protein-containing complex assembly | 53 | 25.43 | 2.08 | 3.11E-06 | 6.12E-04 |
| heterocycle metabolic process | 134 | 87.38 | 1.53 | 3.65E-06 | 6.97E-04 |
| actin cytoskeleton organization | 29 | 10.3 | 2.82 | 3.91E-06 | 7.24E-04 |
| regulation of multicellular organismal process | 73 | 39.92 | 1.83 | 4.65E-06 | 8.36E-04 |
| organic substance biosynthetic process | 131 | 85.32 | 1.54 | 4.87E-06 | 8.53E-04 |
| regulation of localization | 62 | 32.11 | 1.93 | 5.54E-06 | 9.42E-04 |
| cellular aromatic compound metabolic process | 133 | 87.73 | 1.52 | 6.56E-06 | 1.09E-03 |
| protein-containing complex subunit organization | 57 | 28.98 | 1.97 | 7.31E-06 | 1.18E-03 |
| cellular developmental process | 96 | 58.32 | 1.65 | 9.07E-06 | 1.36E-03 |
| gene expression | 103 | 63.86 | 1.61 | 8.69E-06 | 1.37E-03 |
| cellular biosynthetic process | 127 | 83.4 | 1.52 | 8.93E-06 | 1.37E-03 |
| regulation of muscle contraction | 16 | 3.91 | 4.1 | 1.27E-05 | 1.70E-03 |
| regulation of system process | 20 | 5.9 | 3.39 | 1.22E-05 | 1.70E-03 |
| nucleic acid metabolic process | 105 | 65.85 | 1.59 | 1.16E-05 | 1.70E-03 |
| regulation of muscle system process | 16 | 3.91 | 4.1 | 1.27E-05 | 1.74E-03 |
| nucleobase-containing compound metabolic process | 127 | 83.9 | 1.51 | 1.22E-05 | 1.74E-03 |
| cellular macromolecule biosynthetic process | 88 | 52.99 | 1.66 | 1.36E-05 | 1.74E-03 |
| system process | 75 | 42.77 | 1.75 | 1.33E-05 | 1.75E-03 |
| cellular nitrogen compound biosynthetic process | 79 | 46.1 | 1.71 | 1.40E-05 | 1.76E-03 |
| macromolecule biosynthetic process | 89 | 53.56 | 1.66 | 1.44E-05 | 1.78E-03 |
| macromolecule modification | 151 | 104 | 1.45 | 1.48E-05 | 1.79E-03 |
| RNA metabolic process | 82 | 48.52 | 1.69 | 1.58E-05 | 1.81E-03 |
| establishment of localization | 179 | 128.22 | 1.4 | 1.57E-05 | 1.83E-03 |
| developmental process | 183 | 131.49 | 1.39 | 1.56E-05 | 1.85E-03 |
| muscle contraction | 11 | 1.85 | 5.96 | 1.80E-05 | 1.99E-03 |
| muscle system process | 11 | 1.85 | 5.96 | 1.80E-05 | 2.03E-03 |
| cell differentiation | 93 | 57.4 | 1.62 | 2.01E-05 | 2.14E-03 |
| biosynthetic process | 132 | 88.73 | 1.49 | 1.99E-05 | 2.16E-03 |
| regulation of locomotion | 41 | 18.75 | 2.19 | 2.09E-05 | 2.19E-03 |
| anatomical structure development | 173 | 123.54 | 1.4 | 2.14E-05 | 2.21E-03 |
| cell projection organization | 39 | 17.9 | 2.18 | 2.54E-05 | 2.58E-03 |
| regulation of anatomical structure morphogenesis | 29 | 11.51 | 2.52 | 2.66E-05 | 2.66E-03 |
| regulation of cell differentiation | 38 | 17.4 | 2.18 | 3.21E-05 | 3.16E-03 |
| plasma membrane bounded cell projection organization | 37 | 17.12 | 2.16 | 4.84E-05 | 4.69E-03 |
| regulation of striated muscle cell differentiation | 8 | 0.99 | 8.04 | 5.07E-05 | 4.83E-03 |
| nucleic acid-templated transcription | 25 | 9.31 | 2.69 | 5.66E-05 | 5.32E-03 |
| cell development | 52 | 27.71 | 1.88 | 6.19E-05 | 5.65E-03 |
| regulation of developmental process | 64 | 36.66 | 1.75 | 6.15E-05 | 5.70E-03 |
| cell morphogenesis | 27 | 10.94 | 2.47 | 6.72E-05 | 6.04E-03 |
| cellular protein modification process | 137 | 95.83 | 1.43 | 7.58E-05 | 6.45E-03 |
| regulation of cellular process | 316 | 254.89 | 1.24 | 7.71E-05 | 6.47E-03 |
| protein modification process | 137 | 95.83 | 1.43 | 7.58E-05 | 6.53E-03 |
| embryo development ending in birth or egg hatching | 43 | 21.6 | 1.99 | 7.38E-05 | 6.55E-03 |
| RNA biosynthetic process | 25 | 9.66 | 2.59 | 7.52E-05 | 6.57E-03 |
| transcription, DNA-templated | 24 | 9.02 | 2.66 | 8.75E-05 | 7.25E-03 |
| striated muscle cell differentiation | 12 | 2.77 | 4.33 | 9.74E-05 | 7.97E-03 |
| myofibril assembly | 11 | 2.34 | 4.69 | 1.05E-04 | 8.28E-03 |
| actomyosin structure organization | 13 | 3.27 | 3.98 | 1.04E-04 | 8.30E-03 |
| regulation of muscle cell differentiation | 8 | 1.14 | 7.04 | 1.03E-04 | 8.35E-03 |
| regulation of cellular component organization | 62 | 36.02 | 1.72 | 1.11E-04 | 8.60E-03 |
| protein homooligomerization | 15 | 4.33 | 3.46 | 1.20E-04 | 9.20E-03 |
| cellular component assembly involved in morphogenesis | 11 | 2.42 | 4.55 | 1.31E-04 | 9.93E-03 |
| neuron recognition | 10 | 1.99 | 5.03 | 1.36E-04 | 1.02E-02 |
| regulation of transport | 36 | 17.33 | 2.08 | 1.40E-04 | 1.04E-02 |
| neuron projection development | 27 | 11.37 | 2.38 | 1.48E-04 | 1.08E-02 |
| transport | 167 | 123.25 | 1.35 | 1.61E-04 | 1.16E-02 |
| multicellular organism development | 155 | 113.24 | 1.37 | 1.65E-04 | 1.18E-02 |
| DNA-templated transcription, initiation | 12 | 2.98 | 4.02 | 1.75E-04 | 1.21E-02 |
| establishment of localization in cell | 24 | 9.73 | 2.47 | 1.75E-04 | 1.22E-02 |
| protein phosphorylation | 58 | 33.32 | 1.74 | 1.73E-04 | 1.22E-02 |
| microtubule-based process | 38 | 19.11 | 1.99 | 1.89E-04 | 1.28E-02 |
| protein complex oligomerization | 15 | 4.55 | 3.3 | 1.89E-04 | 1.29E-02 |
| positive regulation of muscle cell differentiation | 7 | 0.92 | 7.58 | 2.00E-04 | 1.33E-02 |
| positive regulation of striated muscle cell differentiation | 7 | 0.92 | 7.58 | 2.00E-04 | 1.34E-02 |
| muscle cell differentiation | 13 | 3.55 | 3.66 | 2.10E-04 | 1.35E-02 |
| neuron development | 29 | 12.86 | 2.26 | 2.07E-04 | 1.35E-02 |
| establishment or maintenance of cell polarity | 19 | 6.89 | 2.76 | 2.19E-04 | 1.35E-02 |
| system development | 70 | 43.26 | 1.62 | 2.19E-04 | 1.36E-02 |
| cell migration | 24 | 9.87 | 2.43 | 2.07E-04 | 1.36E-02 |
| cell surface receptor signaling pathway | 42 | 22.09 | 1.9 | 2.18E-04 | 1.37E-02 |
| neuron differentiation | 32 | 14.99 | 2.13 | 2.16E-04 | 1.38E-02 |
| positive regulation of cellular process | 98 | 65.64 | 1.49 | 2.27E-04 | 1.39E-02 |
| regulation of actin cytoskeleton organization | 16 | 5.26 | 3.04 | 2.59E-04 | 1.52E-02 |
| positive regulation of locomotion | 22 | 8.67 | 2.54 | 2.55E-04 | 1.52E-02 |
| plasma membrane bounded cell projection morphogenesis | 23 | 9.38 | 2.45 | 2.55E-04 | 1.53E-02 |
| positive regulation of biological process | 119 | 83.54 | 1.42 | 2.72E-04 | 1.54E-02 |
| neuron projection morphogenesis | 23 | 9.38 | 2.45 | 2.55E-04 | 1.54E-02 |
| cell motility | 25 | 10.44 | 2.39 | 2.72E-04 | 1.56E-02 |
| cell recognition | 10 | 2.2 | 4.54 | 2.69E-04 | 1.57E-02 |
| localization of cell | 25 | 10.44 | 2.39 | 2.72E-04 | 1.57E-02 |
| muscle cell development | 11 | 2.7 | 4.07 | 2.94E-04 | 1.64E-02 |
| striated muscle cell development | 11 | 2.7 | 4.07 | 2.94E-04 | 1.65E-02 |
| cell projection morphogenesis | 23 | 9.52 | 2.42 | 3.01E-04 | 1.66E-02 |
| taxis | 28 | 12.64 | 2.21 | 3.23E-04 | 1.77E-02 |
| organic cyclic compound biosynthetic process | 50 | 28.2 | 1.77 | 3.32E-04 | 1.79E-02 |
| regulation of postsynaptic membrane potential | 10 | 2.27 | 4.4 | 3.32E-04 | 1.80E-02 |
| supramolecular fiber organization | 22 | 8.95 | 2.46 | 3.44E-04 | 1.83E-02 |
| chemotaxis | 26 | 11.58 | 2.25 | 3.56E-04 | 1.88E-02 |
| striated muscle myosin thick filament assembly | 6 | 0.71 | 8.45 | 3.78E-04 | 1.98E-02 |
| regulation of actin filament-based process | 16 | 5.47 | 2.93 | 3.81E-04 | 1.98E-02 |
| cell-cell signaling | 38 | 19.89 | 1.91 | 3.90E-04 | 2.00E-02 |
| aromatic compound biosynthetic process | 46 | 25.64 | 1.79 | 3.89E-04 | 2.01E-02 |
| embryo development | 60 | 36.23 | 1.66 | 4.27E-04 | 2.17E-02 |
| tissue development | 25 | 11.08 | 2.26 | 4.46E-04 | 2.21E-02 |
| cell morphogenesis involved in differentiation | 22 | 9.16 | 2.4 | 4.38E-04 | 2.21E-02 |
| cellular localization | 81 | 52.92 | 1.53 | 4.46E-04 | 2.23E-02 |
| biological adhesion | 17 | 6.18 | 2.75 | 4.72E-04 | 2.30E-02 |
| cell adhesion | 17 | 6.18 | 2.75 | 4.72E-04 | 2.32E-02 |
| cell morphogenesis involved in neuron differentiation | 21 | 8.6 | 2.44 | 5.02E-04 | 2.43E-02 |
| myosin filament organization | 6 | 0.78 | 7.68 | 5.52E-04 | 2.55E-02 |
| mesenchyme development | 7 | 1.14 | 6.16 | 5.31E-04 | 2.55E-02 |
| regulation of multicellular organismal development | 45 | 25.36 | 1.77 | 5.38E-04 | 2.56E-02 |
| establishment of cell polarity | 14 | 4.55 | 3.08 | 5.57E-04 | 2.56E-02 |
| myosin filament assembly | 6 | 0.78 | 7.68 | 5.52E-04 | 2.57E-02 |
| anterograde trans-synaptic signaling | 30 | 14.63 | 2.05 | 5.50E-04 | 2.59E-02 |
| chemical synaptic transmission | 30 | 14.63 | 2.05 | 5.50E-04 | 2.61E-02 |
| heterocycle biosynthetic process | 46 | 26 | 1.77 | 5.99E-04 | 2.73E-02 |
| regulation of axon extension | 11 | 2.98 | 3.69 | 6.03E-04 | 2.73E-02 |
| cell part morphogenesis | 23 | 9.87 | 2.33 | 6.23E-04 | 2.78E-02 |
| axon guidance | 19 | 7.25 | 2.62 | 6.23E-04 | 2.80E-02 |
| negative regulation of cellular process | 78 | 51.29 | 1.52 | 6.31E-04 | 2.80E-02 |
| neuron projection guidance | 19 | 7.32 | 2.6 | 6.50E-04 | 2.86E-02 |
| cellular protein-containing complex assembly | 38 | 20.39 | 1.86 | 6.62E-04 | 2.88E-02 |
| regulation of cell development | 26 | 11.93 | 2.18 | 6.60E-04 | 2.89E-02 |
| nucleobase-containing compound biosynthetic process | 42 | 23.37 | 1.8 | 6.83E-04 | 2.95E-02 |
| regulation of extent of cell growth | 11 | 3.05 | 3.6 | 7.13E-04 | 3.05E-02 |
| anatomical structure morphogenesis | 60 | 37.3 | 1.61 | 7.22E-04 | 3.07E-02 |
| axonogenesis | 20 | 8.24 | 2.43 | 7.34E-04 | 3.08E-02 |
| peptidyl-amino acid modification | 53 | 31.83 | 1.67 | 7.30E-04 | 3.08E-02 |
| regulation of hydrolase activity | 33 | 16.84 | 1.96 | 7.46E-04 | 3.11E-02 |
| peptidyl-serine modification | 23 | 10.16 | 2.26 | 7.69E-04 | 3.18E-02 |
| regulation of sarcomere organization | 6 | 0.85 | 7.04 | 7.81E-04 | 3.20E-02 |
| positive regulation of sarcomere organization | 6 | 0.85 | 7.04 | 7.81E-04 | 3.22E-02 |
| axon development | 22 | 9.31 | 2.36 | 8.07E-04 | 3.26E-02 |
| intracellular signal transduction | 47 | 27.28 | 1.72 | 8.06E-04 | 3.27E-02 |
| regulation of cell growth | 13 | 4.19 | 3.1 | 8.18E-04 | 3.28E-02 |
| reproduction | 80 | 53.63 | 1.49 | 8.34E-04 | 3.33E-02 |
| trans-synaptic signaling | 30 | 14.7 | 2.04 | 8.48E-04 | 3.36E-02 |
| synaptic signaling | 30 | 15.13 | 1.98 | 1.01E-03 | 3.96E-02 |
| positive regulation of cell differentiation | 18 | 7.03 | 2.56 | 1.01E-03 | 3.97E-02 |
| protein-containing complex localization | 15 | 5.47 | 2.74 | 1.02E-03 | 3.97E-02 |
| microtubule-based transport | 12 | 3.77 | 3.19 | 1.05E-03 | 4.02E-02 |
| transport along microtubule | 12 | 3.77 | 3.19 | 1.05E-03 | 4.04E-02 |
| generation of neurons | 38 | 20.74 | 1.83 | 1.07E-03 | 4.10E-02 |
| macromolecule localization | 80 | 54.13 | 1.48 | 1.12E-03 | 4.23E-02 |
| chemical synaptic transmission, postsynaptic | 8 | 1.78 | 4.5 | 1.14E-03 | 4.26E-02 |
| excitatory postsynaptic potential | 8 | 1.78 | 4.5 | 1.14E-03 | 4.29E-02 |
| neurogenesis | 38 | 20.96 | 1.81 | 1.14E-03 | 4.31E-02 |
| regulation of cytoskeleton organization | 22 | 9.87 | 2.23 | 1.18E-03 | 4.36E-02 |
| phosphorylation | 72 | 47.67 | 1.51 | 1.20E-03 | 4.42E-02 |
| response to stimulus | 237 | 192.23 | 1.23 | 1.22E-03 | 4.43E-02 |
| positive regulation of hydrolase activity | 21 | 9.02 | 2.33 | 1.21E-03 | 4.43E-02 |
| catabolic process | 88 | 60.88 | 1.45 | 1.28E-03 | 4.63E-02 |
| peptidyl-serine phosphorylation | 22 | 10.02 | 2.2 | 1.33E-03 | 4.78E-02 |
